# Dietary Supplementation with Black Raspberries Altered the Gut Microbiome Composition in a Mouse Model of Colitis-Associated Colorectal Cancer, although with Differing Effects for a Healthy versus a Western Basal Diet

**DOI:** 10.3390/nu14245270

**Published:** 2022-12-10

**Authors:** Daphne M. Rodriguez, Korry J. Hintze, Giovanni Rompato, Arnaud J. Van Wettere, Robert E. Ward, Sumira Phatak, Canyon Neal, Tess Armbrust, Eliza C. Stewart, Aaron J. Thomas, Abby D. Benninghoff

**Affiliations:** 1 Department of Animal, Dairy and Veterinary Sciences, 4815 Old Main Hill, Utah State University, Logan, UT 84322, USA; 2 Department of Nutrition, Dietetics and Food Sciences, 8700 Old Main Hill, Utah State University, Logan, UT 84322, USA

**Keywords:** black raspberry, Western diet, inflammation, colitis, colon tumorigenesis, microbiome, alpha diversity, beta diversity

## Abstract

Black raspberries (BRB) are rich in anthocyanins with purported anti-inflammatory properties. However, it is not known whether dietary supplementation would ameliorate Western-diet enhanced gut inflammation and colon tumorigenesis. We employed a mouse model of colitis-associated colorectal cancer (CAC) to determine the effects of dietary supplementation with 5 to 10% (*w*/*w*) whole, freeze-dried BRB in male C57BL/6J mice fed either a standard healthy diet (AIN93G) or the total Western diet (TWD). In a pilot study, BRB suppressed colitis and colon tumorigenesis while also shifting the composition of the fecal microbiome in favor of taxa with purported health benefits, including *Bifidobacterium pseudolongum*. In a follow-up experiment using a 2 × 2 factorial design with AIN and TWD basal diets with and without 10% (*w*/*w*) BRB, supplementation with BRB reduced tumor multiplicity and increased colon length, irrespective of the basal diet, but it did not apparently affect colitis symptoms, colon inflammation or mucosal injury based on histopathological findings. However, BRB intake increased alpha diversity, altered beta diversity and changed the relative abundance of Erysipelotrichaceae, Bifidobacteriaceae, Streptococcaceae, Rikenellaceae, Ruminococcaceae and Akkermansiaceae, among others, of the fecal microbiome. Notably, changes in microbiome profiles were inconsistent with respect to the basal diet consumed. Overall, these studies provide equivocal evidence for in vivo anti-inflammatory effects of BRB on colitis and colon tumorigenesis; yet, BRB supplementation led to dynamic changes in the fecal microbiome composition over the course of disease development.

## 1. Introduction

Inflammatory bowel disease (IBD) encompasses a group of disorders characterized by chronic intestinal inflammation, including ulcerative colitis (UC) and Crohn’s disease (CD). IBD is an idiopathic disorder, with causative factors linked to heredity, genetics and environmental risk factors. While the risk factors for both conditions are similar [1,2], UC is characterized by inflammation limited to the colon, whereas CD may involve any part of the digestive tract. As of 2015, an estimated 3.1 million adults in the United States had been diagnosed with IBD, according to the Centers for Disease Control and Prevention [3], and recent reports indicate that global prevalence of IBD has increased markedly in the past thirty years [4].

Patients diagnosed with IBD have a two to six times higher risk of developing colorectal cancer (CRC) compared to healthy individuals [5], and they tend to be affected at a younger age than those who develop sporadic CRC [6]. The National Cancer Institute estimated 149,500 new cases of colorectal cancer in 2021, placing this disease as the fourth most diagnosed cancer in the United States [7]. Colorectal cancer is estimated to be the third leading cause of cancer death in the United States, with approximately 52,980 deaths reported in 2021. The risk of developing colitis-associated colorectal cancer (CAC) begins to increase eight to ten years after the onset of inflammation and increases with prolonged intestinal inflammation [6]. Other factors contributing to the risk of developing CRC include African American decent, male sex, metabolic status, excessive consumption of red or processed meat, alcohol and/or tobacco habits and chronic inflammation associated with IBD, among others [8]. However, IBD and CRC risk can be reduced with changes in lifestyle, such as regular physical activity, consumption of a prudent diet and intake of non-steroid anti-inflammatory drugs in elderly populations [6].

Diet is a major risk factor affecting the pathogenesis of IBD and CRC. The typical American diet is energy dense and nutrient poor, characterized by high proportions of red meat, animal fat and sugar coupled with low fiber intake [9]. Chronic deficiencies of essential micronutrients typified by the Western dietary pattern can lead to chronic disease by disrupting metabolic and other biological pathways [10]. To better understand the contribution of a Western dietary pattern to the development of colon inflammation and CAC, our group previously developed a novel basal diet that emulates typical American nutrient intakes, including macro- and micronutrient profiles, on an energy-density basis for rodents [11]. In repeated studies using this total Western diet (TWD), we have shown that chronic consumption of this Western nutrition profile in mice markedly exacerbates colitis and promotes development of colorectal tumors, likely through activation of pro-inflammatory and aberrant immune response pathways in the colon mucosa [12], although without triggering systemic inflammation or metabolic syndrome [13].

Anthocyanins are water-soluble compounds that impart blue, red and purple colors to certain fruits and vegetables, such as cranberries, blueberries, blackberries, strawberries, black currant, bilberry and purple corn [14]. Anthocyanins are characterized as either the anthocyanin glycosides (e.g., cyanidin-3-glucoside) or the sugar-free anthocyanidin aglycone. Because anthocyanins are poorly absorbed in the gastrointestinal tract, a large fraction of these bioactive chemicals reach the colon and are available for microbial metabolism to generate the more bioavailable metabolite protocatechuic acid, which has reported antioxidant, anti-diabetic, anti-proliferative and pro-apoptotic capabilities [15]. Moreover, researchers have reported two anthocyanidins, delphinidin and cyanidin, exhibiting cytotoxicity toward metastasizing colorectal cancer cells via oxidative stress [16].

The black raspberry (*Rubus occidentalis*) is a good source of vitamins A, C, E, calcium, folic acid and fiber, as well as an abundant source of bioactive phytochemicals, including anthocyanins, ellagic acid, ferulic acid, ellagitannins and other bioflavonoids [17]. Anthocyanins comprise the most abundant polyphenol class in black raspberries (BRB), with an estimated total concentration of 669 mg anthocyanin per 100 g berries [18]. The average American is estimated to consume 12.5 mg of anthocyanins a day [18]. Black raspberries (BRB) contain phytochemicals regularly metabolized by host microbes, generating secondary metabolites that exhibit antioxidant, antiproliferation and pro-apoptotic properties [19,20]. Cyanidin-3-*O*-glucoside is the most abundant anthocyanin present in fruits and veggies [21]. In one report, the consumption of a diet enriched with 5% BRB (*w*/*w*) resulted in reduced cell proliferation, inflammation and angiogenesis and increased apoptosis in a rat model of esophageal cancer [22]. BRB consumption has been shown to modulate inflammatory pathways in the colon by reducing expression of TNF and IL-1β, as well as other key mediators of inflammation, such as NFκB and COX-2 [23]. In addition, BRB promotes the production of short-chain fatty acids (SCFAs) via microbe fermentation, as seen in rats [24]. Various studies have also observed a shift in the gut microbiota composition due to BRB consumption promoting beneficial gut bacteria growth, such as *Akkermansia muciniphila*, *Bifidobacterium spp.* and *Lactobacillus spp.,* while inhibiting pathogenic strains, such as *Helicobacter pylori* [25,26].

When considering the role of diet in modulating gut inflammation and development of intestinal cancer, one cannot ignore the potential involvement of the gut microbiome, that is, the collection of thousands of diverse organisms within the intestinal tract that help maintain gut homeostasis [27]. However, dysbiosis can occur when certain taxa become aberrantly abundant, or pathogenic bacteria are present. Importantly, the gut microbiome modulates physiological functions related to cancer development, including inflammation, cell proliferation, apoptosis and angiogenesis. In the pathogenesis of IBD, a shift in the microbiota population, triggered by a combination of genetic and environmental factors, leads to dysregulation of the immune system, disruption of the epithelial barrier, increased production of pro-inflammatory and pro-tumorigenic cytokines, metabolic activation of various mutagens, loss of protective bacteria species and accumulation of opportunistic pathobionts (reviewed in Refs [28,29]). The gut microbiomes of IBD patients are distinct from healthy controls, with consistent reduced gut microbial biomass, decreased diversity and richness of the microbial community and/or altered relative abundance of members of the dominant phyla, Firmicutes (synonym Bacillota) and Bacteroidetes (synonym Bacteroidota) [30,31,32,33]. In a longitudinal study assessing the structure and function of the mouse microbiome during active colitis and during a subsequent period of recovery, Schwab et al. [34] determined that changes in the microbiome caused by exposure to the colonic irritant dextran sodium sulfate (DSS) were temporary, with functional recovery of the metagenome occurring shortly after cessation of DSS exposure and the taxonomic composition returning within 25 days. These findings illustrate the remarkable ability of the gut microbiome to recover host-microbiota homeostasis after gut injury in this chemically induced animal model of UC.

Although there is evidence pointing to health benefits of BRB consumption for suppression of colitis and colon tumor development, it is not known whether BRB intake in the context of a Western diet would affect gut inflammation and CAC or whether BRB would alter the composition of the microbiome. Thus, the objective of this study was to determine the effects of dietary intervention with whole freeze-dried black raspberries on the dynamic composition of the gut microbiome, inflammation status and colon tumorigenesis in mice fed a Western diet. Based on prior evidence for protective effects of BRB reported in the literature, we hypothesized a dietary supplementation with BRB would improve recovery from colon injury and prevent progression to CAC, and this effect would be more pronounced in mice consuming a Western diet. We also hypothesized that consumption of BRB would result in changes to the gut microbiota composition, shifting the population in favor of commensal species that promote gut homeostasis.

## 2. Materials and Methods

### 2.1. Chemicals and Reagents

Azoxymethane (AOM) was purchased from Sigma-Aldrich (St. Louis, MO, USA; CAS No. 25843-45-2). Dextran sodium sulfate (DSS; reagent grade at mol. wt. ~40 kDa) was obtained from Thermo Fisher Scientific (Waltham, MA, USA). All other chemicals were obtained from general laboratory suppliers at reagent grade. Other reagents and kits are described below.

### 2.2. Animals and Experimental Diets

The Utah State University Institutional Animal Care and use Committee approved all procedures for the handling and treatment of mice used for this study (protocol 2818). Mice were housed in a specific pathogen-free vivarium in the Laboratory Animal Research Center (LARC) at Utah State University, an AAALAC-approved facility. To be consistent with previous work on the role of the TWD in CAC [12,13,35,36], male C57BL/6J mice were obtained from Jackson Laboratories (Bar Harbor, ME) at five weeks of age. Mice were group-housed in sterile microisolator cages with Bed-o’Cobs^®^ ¼ bedding (Andersons, Cincinnati, OH, USA) supplied with HEPA-filtered microisolator cages in an IVC Air Handling Solutions ventilated housing system (Tecniplast, Buguggiate, Italy). Mice were maintained in a 12:12 h dark:light cycle, with 50% humidity and in a specific pathogen-free vivarium with temperature ranging from 18 to 23 °C. Following one week of quarantine, mice were randomized and allocated to one of four experimental groups, as outlined below. Ear notching was performed to allow for repeated individual mouse weight measurements weekly. Mice were provided with autoclaved drinking water *ad libitum* throughout the study.

Experimental diets were formulated by Envigo (Hackensack, NJ; formerly Harlan-Teklad), as outlined in Table S1, obtained from the vendor as one lot and maintained at 4 °C for the duration of the study. The two basal diets included AIN93G (AIN, cat. no. TD.160421), formulated to promote rodent health with energy density of 3.8 kcal/g, or the total Western diet (TWD, cat. no. TD.160422, formulation previously published [11]) with energy density of 4.4 kcal/g, designed to emulate typical U.S. intakes of macro- and micronutrients on an energy density basis. Fresh food was provided twice a week, and food consumption was monitored at each change (including accounting for spillage into the cage).

Black raspberry (*Rubus occidentalis*, Munger variety) powder (BRB) was obtained from Berri Health (Corvallis, OR, USA). This powder consists of 6.94% (*w*/*w*) total phenolics with 3.72% (*w*/*w*) total anthocyanins with cyanidin-3-rutinoside as the dominant form and small quantities of cyanidin-3-glucoside, cyanidin-3-xyloside and cyanidin-3-arabinoside (via Certificate of Analysis and personal communication, J. Stephen Dunfield, President, Berri Products LLC). Other phenolics and flavonoids include caffeic acid, ellagic acid and quercetin. AIN and TWD basal diets were supplemented with 5 or 10% (*w*/*w*) BRB, with adjustments made to equalize total carbohydrates with respect to the appropriate basal diet. Given an estimated daily food intake of 3.5 g per day per mouse (equating to 13.3 or 15.1 kcal/day for AIN and TWD diets, respectively), this concentration of BRB will deliver approximately 7 to 13 mg anthocyanins per mouse per day, for 5 and 10% supplementation levels, respectively. These concentrations correspond to energy densities of 489 and 978 µg/kcal for the AIN basal diet or 432 and 864 µg/kcal for the TWD basal diet for the high and low BRB concentration, respectively. The black raspberry Munger variety was estimated to provide 394 mg total anthocyanins per 100 g serving, or 190 µg/kcal for one serving per day with a total caloric intake of 2070 kcal/day (U.S. average). Thus, the 5% and 10% BRB supplementation for the mouse diets would approximate two or five servings, respectively, of whole BRB fruit per day on an energy density basis.

### 2.3. Experiment Designs

#### 2.3.1. Pilot Study (Experiment A)

A pilot study was performed to explore the potential benefit of dietary supplementation with black raspberries in mice fed the TWD. Experimental groups included 1) AIN93G basal diet alone as negative control (AIN), 2) TWD basal diet alone as positive control for promotion of colitis and CAC (TWD), 3) TWD supplemented with 5% (*w*/*w*) BRB (TWD + 5%BRB) and 4) TWD supplemented with 10% (*w*/*w*) BRB (TWD + 10%BRB). At five weeks of age, mice were assigned to these diet groups using a random block design to equalize group body weight at the start of the experiment (*n* = 32 per diet group) (Figure S1a). Mice were provided either the AIN diet (group 1) or the TWD diet (groups 2–4) for seven days, at which time the BRB-supplemented diets were introduced (groups 3–4). On day 21, all mice were dosed *i.p.* with 10 mg/kg AOM prepared in sterile PBS and provided 1% (*w*/*v*) DSS, a colonic irritant, in their drinking water for 10 days, followed by plain drinking water for the remainder of the study. On experiment days 33 and 45, mice were temporarily placed in new cages blinded to treatment, and then, the disease activity index (DAI) was determined, as previously described [12]. On day 105, body composition was determined for all mice using an MRI scan (EchoMRI-700; EchoMRI, Houston, TX, USA). On day 112, mice were euthanized by CO_2_ asphyxiation and necropsied. Colons were isolated (*n* = 23 to 26 per group), flushed with PBS, cut open longitudinally and stored at 4 °C in 70% (*v*/*v*) ethanol until further assessment of colon tumors, as described previously [12]. A randomly selected subset of colon tissues (*n* = 6 per group) was preserved in 10% phosphate-buffered formalin for histopathological classification of cancer stage.

#### 2.3.2. BRB Supplementation with Standard and Western Basal Diets (Experiment B)

To determine the effect of BRB supplementation on colitis and CAC in mice fed either a standard diet or a Western diet, a 2 × 2 factorial study design was used with *basal diet* and *BRB supplementation* as the two main factors with the following experimental groups: (1) AIN control (AIN/CON), (2) AIN + 10% BRB (AIN/BRB), (3) TWD control (TWD/CON) and (4) TWD + 10% BRB (TWD/BRB). At five weeks of age, mice were assigned to these diet groups using a random block design to equalize group body weight at the start of the experiment (*n* = 32 per diet group) (Figure S1b). Mice were provided either the AIN diet (groups 1,3) or the TWD diet (groups 2,4) for seven days, at which time BRB-supplemented diets were introduced (groups 3–4). On day 21, all mice were dosed intraperitoneally. with 10 mg/kg AOM prepared in sterile PBS and provided 1% (*w*/*v*) DSS in their drinking water for 10 days, followed by plain drinking water for the remainder of the study. On experiment days 33 and 45, mice were temporarily placed in new cages blinded to treatment, and then, the DAI was determined, as previously described [12]. Additionally, on days 33 and 45, a randomly selected subset of mice (*n* = 9 to 12 per group) were euthanized by CO_2_ asphyxiation, necropsied and their colon tissues preserved in 10% phosphate-buffered formalin for histopathological assessment of epithelial inflammation and mucosal injury by a board-certified veterinary pathologist at the Utah Veterinary Diagnostic Laboratory, as previously described [12]. On day 113, body composition was determined for all mice using an MRI scan (EchoMRI-700). On day 115, the remaining mice were euthanized by CO_2_ asphyxiation and necropsied, as described above. Colons were isolated (*n* = 23 to 26 per group), flushed with PBS, cut open longitudinally and stored at 4 °C in 70% (*v*/*v*) ethanol until further assessment of colon tumors, as described previously [12]. A randomly selected subset of colon tissues (*n* = 6 per group) were preserved in 10% phosphate-buffered formalin for histopathological verification of cancer stage.

### 2.4. Microbiota Profiling by 16S rRNA Sequencing

#### 2.4.1. Microbiome Sequencing (Experiment A)

For experiment A, fresh fecal samples were collected by cage on day 21 (pre-DSS), day 33 (colitis), day 45 (recovery) and day 112 (terminal) and stored at −80 °C until analysis. Obtaining fecal samples on a per cage basis avoided the potential confounding effects of coprophagia among co-housed mice for microbiome analyses. The complete methods for sample preparation, sequencing and data processing are described by Rodriguez et al. [36]. Briefly, the QIAamp DNA Stool Mini Kit (Qiagen, Frederick, MD, USA) was used to isolate DNA from mouse fecal pellets according to the manufacturer’s protocol, with the added step of mechanical disruption with zirconia/silica beads (Thermo Fisher) for 5 min. DNA concentration and sample purity were determined by UV spectrophotometry (NanoDrop 2000, Thermo Fisher). All DNA samples were then diluted to 20 ng/mL in tris-EDTA buffer (TE, pH 8.0). Fecal DNA was then amplified using the Roche High Fidelity dNTP Pack according to the manufacturer’s protocol (Millipore Sigma, St. Louis, MO, USA). Samples were assigned a barcoded primer and a universal primer; the barcoded primers were directed against the V3 region of the 16s rRNA [36]. PCR amplification, product purification and product pooling prior to sequencing were performed as previously described [36]. Samples were sequenced using an Ion Personal Genome Machine (PGM) sequencer with a 318 Chip kit and an Ion PGM Hi-Q View OT2 kit for library preparation (Thermo Fisher) by the USU Center for Integrated BioSystems Genomics Core Laboratory. Sequences were processed using QIIME [37], with mapping of sequences to the GreenGenes OTU database (gg_13_8_otus), as previously described [36]. File S1 provides the resulting count data at the species level.

#### 2.4.2. Microbiome Sequencing (Experiment B)

For experiment B, fresh fecal samples were collected by cage on day 21 (pre-DSS), day 33 (colitis), day 45 (recovery) and day 115 (terminal) and stored at −80 °C until analysis. Because experiment B was performed at a later date than the pilot study, during which time the sequencing instrumentation within the Genomics Core Laboratory had been upgraded, different materials, methods, instrumentation and data processing steps were used for microbiome sequencing of samples. The DNeasy PowerSoil kit (Qiagen) was used for DNA isolation per the supplier’s protocol. DNA concentrations and sample purity were determined using a UV spectrophotometer (NanoDrop 2000). All DNA samples were then diluted to 20 ng/mL in tris-EDTA buffer (TE, pH 8.0). Isolated fecal DNA was amplified using the Platinum HS PCR kit (Thermo Fisher). Forward and reverse primers, 16S-515F and 16S-806R, targeting the V4 region of the 16S rRNA [38] were obtained from Integrated DNA Technologies (Coralville, IA, USA). PCR amplification was performed using the following protocol: 3 min at 94 °C; 35 cycles of 94 °C for 45 s, 50 °C for 60 s, 72 °C for 90 s; final annealing at 72 °C for 10 min; and hold at 4 °C. Next, each sample was assigned a set of barcoding primers, and a second round of PCR amplification was performed using the following protocol: 15 s at 94 °C; 10 cycles at 94 °C for 15 s, 72 °C for 60 s, 72 °C for 90 s; final annealing at 72 °C for 3 min; and hold at 4 °C. Electrophoresis was performed with the amplicons to confirm a product size of 254 bp.

Lastly, the final PCR products were purified using Agencourt AMPure beads (Beckman Coulter, Indianapolis, IN, USA) according to the manufacturer’s instructions. Briefly, PCR products were diluted in the AMPure bead solution, incubated at room temperature for 5 min and then captured using a 96-well magnet for 2 min. The supernatant was removed, and then, the PCR products were rinsed twice with 70% (*v*/*v*) ethanol. DNA was eluted from the beads using TE buffer, and DNA concentrations were reconfirmed by fluorescence spectroscopy (Fluorometer 9300-002, Turner BioSystems, Sunnyvale, CA, USA) using the Quant-IT Picogreen dsDNA Assay (Thermo Fisher). Samples were diluted to 1 ng/μL, pooled and stored at −20 °C until sequencing at the Genomics Core Laboratory. Sequencing was performed using he MiSeq reagent kit v2 for a paired end 500 cycle (2 × 250 bp) (Illumina, San Diego, CA, USA).

Microbiota sequences were processed using QIIME 2 [39] and DADA2 [40]. The DADA2 R package implements the full amplicon workflow (filtering, dereplication, chimera identification, merging paired end reads) and generates an amplicon sequence variant (ASV) table and representative sequences. To assign taxonomy, the Qiime feature-classifier classify-sklearn command was used with a classifier pre-trained for the V4 region, silva-138-99-515-806-nb-classifier.qza, and the most recent release of the Silva database (138 SSU) [41]. File S2 provides the resulting count data at the species level.

### 2.5. Microbiome Sequencing Data Analysis

For all microbiome analyses, the cage was considered the biological unit, which avoided the potential confounding effects of coprophagia of mice that shared housing. For experiment A, the taxonomy and alpha and beta diversity analyses were performed using core_diversity_analyses.py script, as previously described [36]. For experiment B, sequence data were analyzed using Microbiome Analyst Marker Data Profiling module [42], with minimum count of four, a low count filter of 20% prevalence and low variance filter of 10% based on the inter-quantile range. The sequencing libraries were rarefied to the minimum library size with total sum scaling. For experiment A, sequencing data were analyzed for an effect of experimental diet within each time point. For experiment B, data were similarly analyzed for effect of treatment within a time point as well as longitudinally across time points within a treatment. Measures of α-diversity included the number of OTUs or ASVs (total number sequenced), Chao1 richness (number of species represented) and Shannon index (weighted abundance of species present). Beta diversity was determined using unweighted (qualitative measure, which is sensitive to low abundance features) and weighted (accounts for abundance of species) unifrac distance and was represented as principal coordinate plots (PCoA) of the first two coordinates. A permanova *p* value <0.01 for β-diversity was considered statistically significant. Taxonomic relative abundance data were analyzed using metagenomeSeq with a zero-inflated Gaussian fit, and false discovery rate-adjusted *p*-value <0.05 was considered statistically significant. ClustVis was used to perform unsupervised hierarchical cluster analyses using relative abundance data family taxonomic level [43]. Heat trees were constructed to show the relationships among differentially abundant taxa for selected pairwise comparisons. Heat tree analysis leverages the hierarchical structure of taxonomic classifications to quantitatively (using the median abundance) and statistically (using the non-parametric Wilcoxon rank sum test) depict taxonomic differences between microbial communities.

For experiment B only, heat trees were generated for pairwise comparisons by experimental diet or time point. The heat tree analysis leverages the hierarchical structure of taxonomic classifications to quantitatively (using median abundance) and statistically (non-parametric Wilcoxon rank sum test) depict taxonomic differences between microbial communities. Additionally, Tax4Fun was employed for functional potential prediction based on minimum 16S rRNA sequence similarity [44]. The resulting gene abundance tables were processed using the Microbiome Analyst Shotgun Data Profiling module with a minimum count of four, a low count filter of 20% and a low variance filter at 10% based on inter-quartile range. Data were total sum scaled and then analyzed by metagenomeSeq (zero-inflated Gaussian distribution with FDR *p* < 0.05) to identify differentially abundant KEGG orthology terms. The list of significant terms was then subject to pathway association analysis using the *globaltest* algorithm to identify significantly enriched functional pathways (*p* < 0.05). Additionally, to compare the shift in microbiome composition longitudinally in response to BRB intervention, both the taxonomy and functional gene sets were analyzed by non-metric multi-dimensional scaling (NDMS) using the Bray–Curtis dissimilarity method and visualized as principal coordinate plots with the first two coordinates.

### 2.6. Fecal Short-Chain Fatty Acid Analysis

Six acid standards (acetic, propionic, butyric, isobutyric, isovaleric and valeric acids) were prepared, and fecal materials from experiment B were processed for short-chain fatty acid analysis by gas chromatography using a Shimadzu GC2010 equipped with a ZB-FFAP column (30 m × 0.52 mm ID × 1.0 μM film thickness; Phenomenex, Torrance, CA, USA) and a flame ionization detector, as previously described [45].

### 2.7. Other Data Analyses

Statistical analyses for tumor incidence were performed using Fisher’s exact test, followed by a Bonferroni adjustment to correct for multiple testing (Prism v. 8, GraphPad Software, San Diego, CA). Other data were analyzed using a generalized linear mixed model (GLMM) with cage as a nested, random factor using the restricted maximum likelihood (REML) estimation and the Tukey HSD post hoc test for multiple comparisons (JMP v.16.2.0, SAS Institute, Cary, NC, USA). For experiment A, the main effect of treatment was determined within each time point. For experiment B, the main effects of basal diet, BRB supplementation and diet*BRB interaction were determined within each time point. Suspected outliers were verified using the robust outlier test (ROUT) with a conservative Q value of 1% (Prism), meaning that there is a ≤1% chance of excluding a data point as an outlier in error. Data that did not meet the equal variance assumption were log_10_ or square root transformed. For data that were not normally distributed or for which a transformation did not equalize variance, a non-parametric Steel–Dwass test was employed (JMP) to assess the main effects of diet and BRB intervention (no interaction test possible). However, if the results of the non-parametric Steel–Dwass tests were not different from the original GLMM analyses with respect to significant outcomes, the original GLMM test results are reported (with interactions for Experiment B) because the mixed model accounts for potential cage effects. A significant effect of the test variable was inferred when the adjusted *p* value was <0.05. Food and energy intakes were assessed on a per cage basis.

## 3. Results

### 3.1. Pilot Study with BRB Supplementation (Experiment A)

#### 3.1.1. Food and Energy Intakes, Body Weight, Lean and Fat Mass, Glucose Tolerance (Experiment A)

In the pilot study, mice fed the TWD basal diet consumed fewer grams of food compared to those mice provided the AIN diet, although their energy intakes were not different (Figure S2a,b), as has been observed previously [12]. The energy intake was significantly greater for mice provided TWD supplemented with either 5% or 10% BRB, corresponding to a significant concentration-dependent increase in final body weight (Figure S2c) attributed to a significant increase in fat mass (Figure S2e). However, the increase in body weight and altered body composition were not associated with impaired glucose metabolism, as glucose tolerance was not affected by BRB supplementation (Figure S2f).

#### 3.1.2. Symptoms of Colitis and Colon Tumor Outcomes (Experiment A)

**Figure 1:**
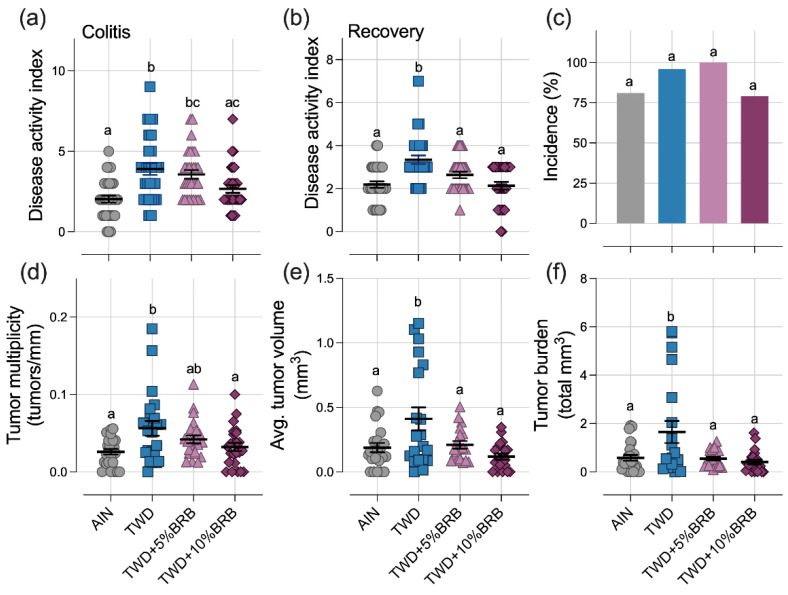
Dietary supplementation with 5 to 10% BRB suppressed symptoms of colitis and colon tumorigenesis in mice fed TWD (experiment A). (**a**,**b**) Disease activity index (DAI) score during active colitis on day 33 (**a**) and recovery from gut injury on day 45 (**b**). (**c**) Incidence of colon tumors shown as the percent of mice with tumors. (**d**) Colon tumor multiplicity (number of tumors per mm colon length). (**e**) Average tumor volume. (**f**) Tumor burden (total volume). For (**a**,**b**) and (**d**–**f**), data are shown as individual values with mean ± SE. Different letters indicate that treatment groups are significantly different (*p* < 0.05), as determined by statistical methods outlined in the Materials and Methods section.

Symptoms of colitis were assessed on experiment days 33 and 45 at the colitis and recovery time points, respectively (Figure S1a). As expected, consumption of the TWD increased the DAI score during active colitis as compared to mice fed the AIN diet, and this response persisted to the recovery time point twelve days later (Figure 1a,b). Addition of BRB to the diet reduced TWD-enhanced colitis symptoms in a concentration-dependent manner, most notably by the recovery time point, at which time the symptoms of colitis in mice fed TWD with either 5% or 10% BRB were not different from mice fed the AIN diet.

At the end of the study, colon tumor incidence was not significantly different among the experimental groups (Figure 1c). However, as anticipated, mice fed TWD had more colon tumors, larger tumors and a significantly higher tumor burden than their AIN-fed counterparts (Figure 1d–f). Remarkably, supplementation of the TWD with 5% or 10% BRB suppressed colon tumorigenesis, leading to fewer and smaller tumors and an overall reduced tumor burden, similar to mice provided the basal AIN diet (Figure 1d–f).

#### 3.1.3. Microbiome Assessment (Experiment A)

A total of 9.9 × 10^6^ amplicons were sequenced. After filtering for length, quality, and abundance and inspection for chimeras, 7.2 × 10^6^ sequences were assigned to OTUs using the pick_open_ref_otus command (GreenGenes database gg_13_8_otus) for an average of 62,073 sequences per sample assigned to 1415 OTUs. The sequencing depth for diversity analyses was set to ~5500 sequences (Figure S3).

Dietary supplementation with 5% or 10% black raspberry shifted the composition of the gut microbiome, primarily during the active colitis phase in this disease model (Figures S4 and S5, File S3). At the phylum taxonomic level, the relative abundance of Actinobacteria was lower in mice fed the TWD basal diet at 13.6% compared to those fed the AIN basal diet at 20% during active colitis, although this difference was not statistically different. BRB supplementation significantly elevated the abundance of Actinobacteria in the fecal microbiome from 13.6% in TWD-fed mice to 21 to 23% in TWD-fed mice supplemented with 5% or 10% BRB. This change in Actinobacteria during colitis was largely attributed to shifts in the population of *Bifidobacterium pseudolongum* (*p* = 0.0041 and 0.0028 for 5 and 10% BRB diets, respectively) in the Bifidobacteriaceae family (Figure S5b). However, also within the Actinobacteria phylum, the abundance of the family Coriobacteriaceae was suppressed with BRB supplementation during both active colitis (*p* = 0.0587 and 0.0048 for 5 and 10% BRB, respectively) and at the terminal time point (BRB treatments combined, *p* = 0.0419). Within the Bacteroidetes phylum (recently renamed Bacteroidota), a substantial decrease in Bacteroidaceae was observed, from 2.4% of the fecal microbiome in mice provided TWD only to just 0.23 to 0.32% in mice fed TWD + 5% or 10% BRB, respectively (*p* = 1.68 × 10^−7^ and 6.14 × 10^−3^, respectively; Figure S5b). Within the Firmicutes phylum (recently renamed Bacillota), the relative abundance of Erysipelotrichaceae was elevated during colitis in mice provided TWD + 5%BRB compared to those fed TWD only (*p* = 3.25 × 10^−9^), whereas the Ruminococcaceae family was less abundant in mice fed either TWD + 5% or 10% BRB compared to TWD only (*p* = 0.0052 or = 0.0023, respectively). Through recovery and to the terminal time point, most of the BRB-induced changes in the microbiome population had resolved with few persistent shifts (e.g., Ruminococcaceae) with a few additional taxa responsive to the TWD + 5% BRB treatment compared to TWD only (e.g., Dehalobacteriaceae, Lachnospiraceae, Peptostreptococcaceae and Rikenellaceae) (File S3). The ratio of Firmicutes:Bacteroidetes was variable across treatment groups and time points, with the only apparent significant difference noted during colitis when comparing the TWD versus the TWD + 5% BRB diet group (*p* = 0.0079) (Figure S5c).

**Figure 2:**
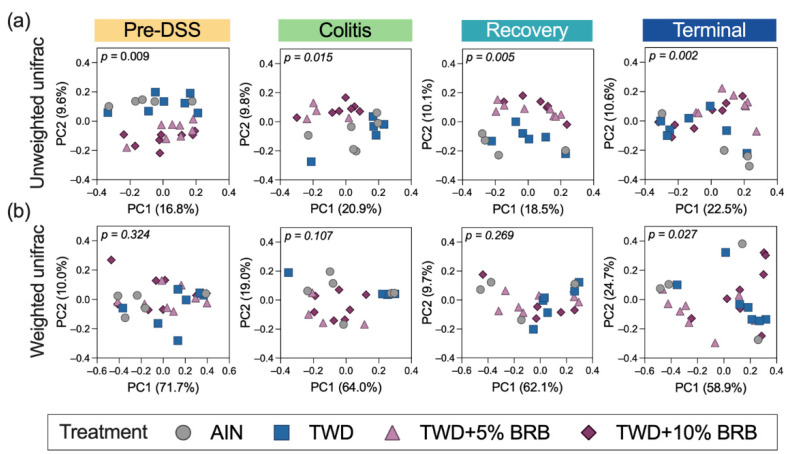
Beta diversity of mouse microbiomes at each experimental time point (experiment A). Principal coordinate plots depicting fecal microbiome beta diversity using (**a**) unweighted or (**b**) weighted unifrac distances are shown with the two components. The variation attributed to PC1 and PC2 along with the permanova *p*-values are provided for each plot.

Alpha diversity was determined as the number of observed OTUs, the Chao1 index (count of species) and the Shannon index (accounts for proportional abundance) (Figure S6). No significant differences in alpha diversity for observed OTUs or Chao1 index were noted among treatment groups at any of the study time points. However, at the pre-DSS time point, the Shannon alpha diversity was elevated in mice fed TWD + 5%BRB compared to the AIN control (Figure S6c). During active colitis, Shannon alpha diversity was significantly reduced in mice provided TWD + 5% or 10%BRB compared to TWD only. Yet, by the terminal time point, alpha diversity was elevated in mice fed TWD + 5% BRB compared to TWD only but not the higher 10% concentration (Figure S6c). When considering the differences in taxa represented in the fecal microbiome populations, more substantial distinctions were observed particularly for unweighted unifrac beta diversity, a measurement that favors the contribution of rare taxa (Figure 2). Following three weeks of exposure to the experimental diets, a clear separation of treatment groups by BRB supplementation was evident for unweighted unifrac distances (permanova *p* = 0.009). These measurably distinct microbiomes for those mice exposed or not exposed to BRB were evident during active colitis (permanova *p* = 0.015), recovery from gut injury (*p* = 0.005) and colon tumorigenesis at the terminal time point (permanova *p* = 0.002) (Figure 2a). Alternatively weighted unifrac distances, which account for the relative abundance of taxa, were not remarkably different (*p* > 0.01) (Figure 2b).

### 3.2. BRB Supplementation with Standard and Western Basal Diets (Experiment B)

#### 3.2.1. Food and Energy Intake, Body Weight and Composition, Organ and Cecum Weight (Experiment B)

**Figure 3:**
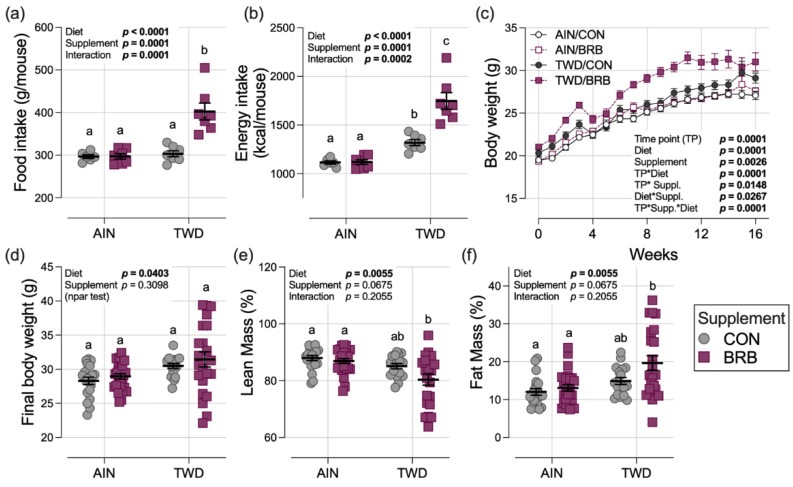
Food and energy intakes, body weight and body composition (experiment B). (**a**,**b**) Estimated total daily food and energy intake per mouse. See Figure S7 for longitudinal food and energy intake data. (**c**) Body weight gain over the study period. (**d**) Final body weight at study end on day 115. (**e**,**f**) Lean and fat mass as percentage of body weight. Data are shown as the individual measurements (except (**c**)) with the mean ± SE (**a**,**b**,**d**–**f**). Inset tables show the statistical model main effects for diet, treatment and their interaction or “npar test” if a non-parametric test was required, and different letters indicate that experimental groups are significantly different (*p* < 0.05), as determined by statistical methods outlined in Materials and Methods.

In control groups, the total food intake for mice fed AIN or TWD basal diets was consistent, leading to a higher overall total energy intake in mice fed the TWD due to that diet’s higher energy density (Figure 3a,b and Figure S7). A significant interaction with the BRB diet was evident (*p* = 0.0002), with food intake at a significantly higher rate for mice fed TWD/BRB compared to their TWD/CON counterparts (*p* < 0.0001), leading to an increased energy intake of 33% compared to AIN/BRB-fed mice (*p* < 0.0001) and AIN/CON (*p* < 0.0001). Although a trend of increased body weight of mice fed the TWD/BRB diet compared to all other groups was evident throughout most of the study period, particularly following the AOM + DSS exposure period days 21–31 (Figure 3c), the final body weights were not significantly different among the experimental groups by day 115 at the study end (Figure 3d). Body composition determined by EchoMRI indicated that lean mass was lower and fat mass was higher, on average, in mice fed TWD/BRB compared to both the AIN/CON and AIN/BRB groups but not compared to mice fed the TWD/CON diet (Figure 3e,f).

Liver weight relative to body weight at the terminal time point was not significantly affected by basal diet or treatment (Figure S8a), whereas kidney weights were slightly increased in mice fed TWD compared to those provided the AIN diet (main effect *p* = 0.0163) (Figure S8b). Relative spleen weights were higher in mice fed the TWD compared to those provided AIN (diet main effect *p* = 0.0005) (Figure S8c), reflecting the higher cancer burden for those animals, as has been noted previously [12]. Interestingly, a strong effect of BRB supplementation was observed for cecum content weight, with the average relative cecum content mass for BRB-exposed mice about 52% greater than the control groups (main effect of treatment *p* = 0.0010) (Figure S8d).

#### 3.2.2. Symptoms of Colitis and Histopathology Scoring (Experiment B)

**Figure 4:**
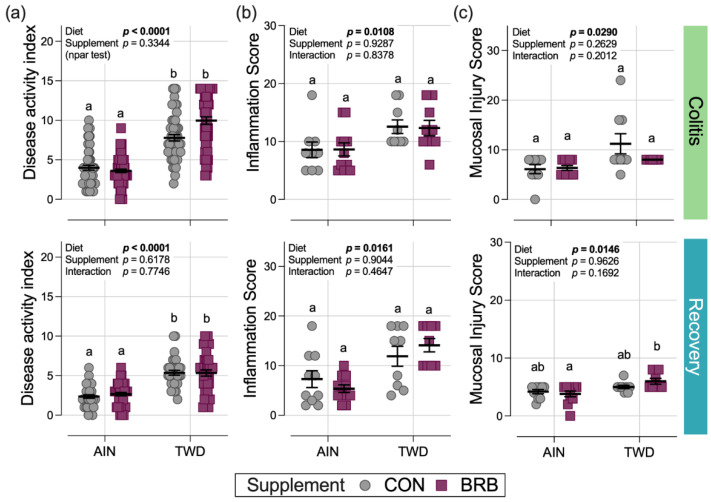
Disease activity index and colon histopathology (experiment B). Scores for the disease activity index (DAI) (**a**), histopathology inflammation score (**b**) and histopathology mucosal injury score (**c**) are shown for active colitis on day 33 and recovery from gut injury on day 45. Data are shown as individual values with mean ± SE. Inset tables provide the statistical model main effects for diet, treatment and their interaction or “npar test” if a non-parametric test was required, and different letters indicate that experimental groups are significantly different (*p* < 0.05), as determined by statistical methods outlined in the Materials and Methods section.

Compared to mice fed the AIN basal diet, consumption of the TWD markedly enhanced the symptoms of colitis as measured by the DAI score during active colitis on day 33 (diet main effect *p* < 0.0001) and continuing through recovery from gut injury at day 45 (diet main effect *p* < 0.0001) (Figure 4a). However, in Experiment B, there was no apparent effect of BRB supplementation on colitis symptoms at either time point (BRB main effect *p =* 0.334 and 0.6178 for colitis and recovery time points, respectively) (Figure 4a). This observation differs from that of the pilot study (Experiment A), in which supplementation of the TWD diet with 10% BRB significantly reduced the DAI at colitis and recovery time points compared to TWD alone (Figure 1a,b).

In a pattern such as that for the DAI score, significant main effects of diet on the colon histopathology inflammation score and mucosa injury scores were noted with higher scores observed in mice fed TWD compared to those provided the AIN diet, but no significant effects of BRB were noted in mice fed either basal diet (Figure 4b,c).

#### 3.2.3. Colon Length and Tumorigenesis (Experiment B)

**Figure 5:**
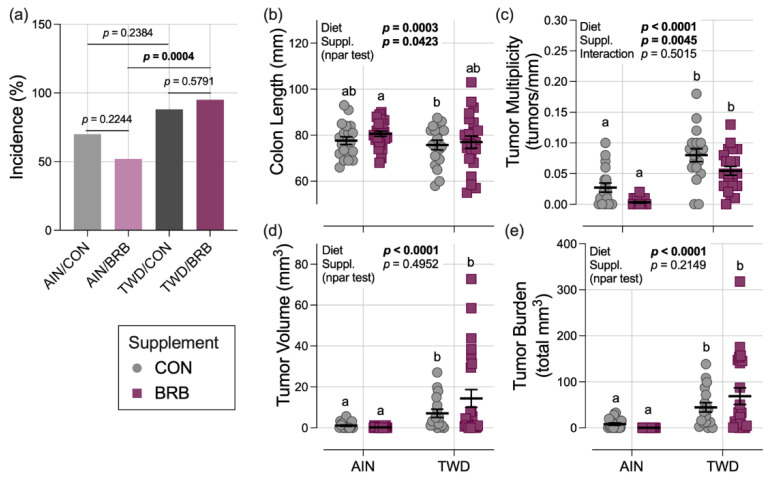
Effect of BRB supplementation on colon length and colon tumorigenesis in mice fed AIN or TWD basal diets (experiment B). (**a**) Incidence of colon tumors shown as the percent of mice with tumors at the terminal time point. P-values from pairwise Fisher exact tests (selected *a priori*) are shown. (**b**) Colon length. (**c**) Colon tumor multiplicity (number of tumors per mm colon length). (**d**) Average tumor volume. (**e**) Tumor burden (total volume). For (**b**–**e**), data are shown as individual values with mean ± SE. Inset tables show the statistical model main effects for diet, treatment and their interaction or “npar test” if a non-parametric test was required, and different letters indicate that experimental groups are significantly different (*p* < 0.05), as determined by statistical methods outlined in the Materials and Methods section.

Colon tumor incidence in mice fed the AIN basal diet was lower in mice supplemented with BRB (52%) compared to their control counterparts (70%), although this difference was not statistically significant (Figure 5a). Similarly, tumor incidence for TWD/CON mice (88%) was no different from the TWD/BRB group (95%). However, incidence in mice provided TWD with BRB was significantly greater than AIN mice also provided BRB (*p* = 0.0004), pointing to an effect of basal diet (Figure 5a).

In mice necropsied on experiment day 115, colons excised from mice fed the TWD were shorter in length by 6.2% at 72.8 mm compared to those fed the AIN diet at 77.6 mm (diet main effect *p* = 0.0003); BRB supplementation significantly increased colon length by 3%, irrespective of basal diet (main effect *p* = 0.0423) (Figure 5b). As was observed repeatedly in this CAC mouse model, consumption of the TWD enhanced colon tumorigenesis, leading to a 4-fold increase in tumor multiplicity, a 15-fold increase in average tumor volume and a 13-fold increase in tumor burden (diet main effect *p* < 0.0001 for all three endpoints) (Figure 5c–e). A significant main effect of BRB treatment on colon tumor multiplicity was observed, with a 46% decline in mice fed BRB compared to controls, regardless of basal diet (*p* = 0.0045). Although tumor multiplicity in the AIN/BRB group was not significantly lower than in their AIN/CON counterparts, a trend of reduced multiplicity was apparent for mice in the TWD/BRB group compared to TWD/CON mice (*p* = 0.0539) (Figure 5c). However, BRB did not effectively reduce colon tumor size or tumor burden (Figure 5d,e).

#### 3.2.4. Dynamics of the Fecal Microbiome in Response to BRB with Differing Basal Diets (Experiment B)

A total of 11.8 × 10^6^ amplicons were sequenced. After filtering for length, quality, and abundance and inspection for chimeras, 7.5 × 10^6^ sequences were assigned to ASVs (Silva database 138 SSU) using the pick_open_ref_otus command for an average of 67,443 sequences per sample assigned to 1415 ASVs. The sequencing depth for diversity analyses was set to 28,909 sequences (Figure S9).

**Figure 6:**
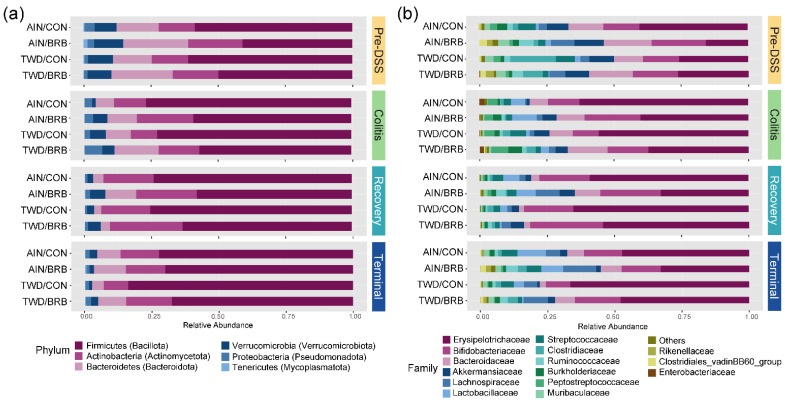
Taxonomic classification of mouse fecal bacteria (experiment B). Data shown are the relative normalized abundance of bacteria annotated to phylum (**a**) or family (**b**) taxonomic levels for the top 15 most abundant taxa for each experimental group for each experimental time point. New phylum level taxonomic designations are indicated in parentheses (**a**).

**Figure 7:**
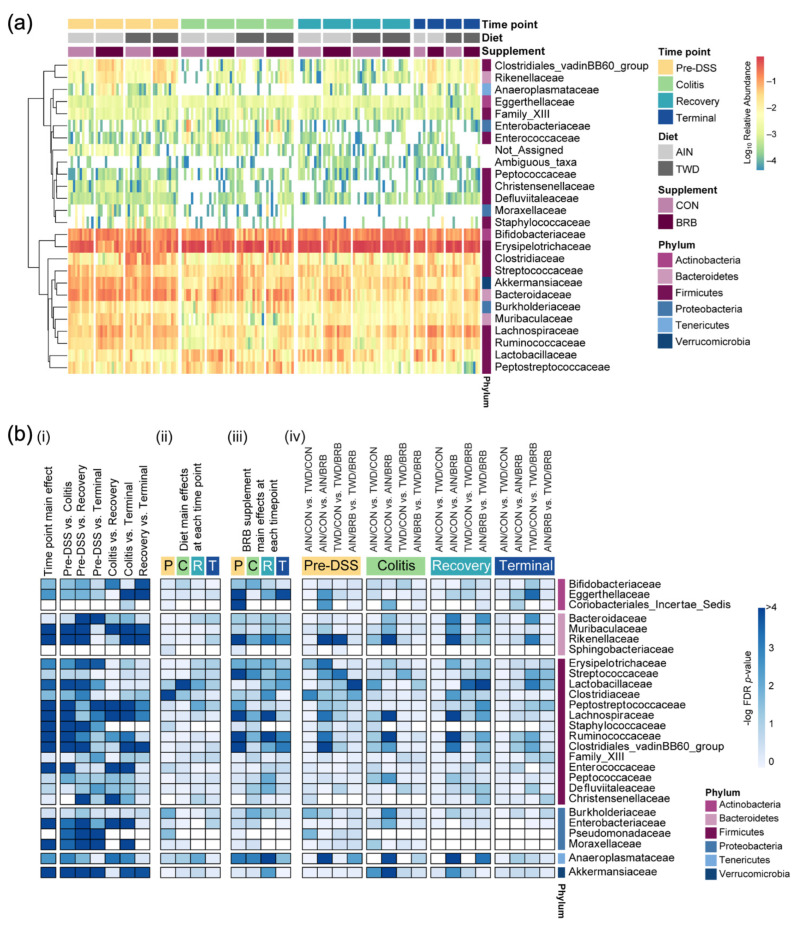
Relative abundance of fecal microbiome at the family taxonomic level with summary of results of metagenomeSeq statistical analyses (experiment B). (**a**) Unsupervised hierarchical cluster analysis shows the log_10_ relative abundance with clustering by taxa using the Euclidean distance with average linkage. (**b**) Summary plot shows the log_10_ FDR-adjusted *p*-values obtained from metagenomeSeq analyses of fecal microbiome profiles. All tests were determined a priori, and complete results are provided in Supplementary File S4. (i) Analyses for main effects of time point and pairwise comparisons across time points, irrespective of basal diet or BRB supplementation. (ii) Analyses for diet main effects, irrespective of BRB supplementation, at each time point. (iii) Analyses for BRB supplementation main effects, irrespective of basal diet, at each time point. (iv) Selected pairwise tests for basal diet and BRB supplement combinations within each study time point. A significant effect was inferred for FDR-adjusted *p*-values <0.05 (increasing blue on the color scale).

**Figure 8:**
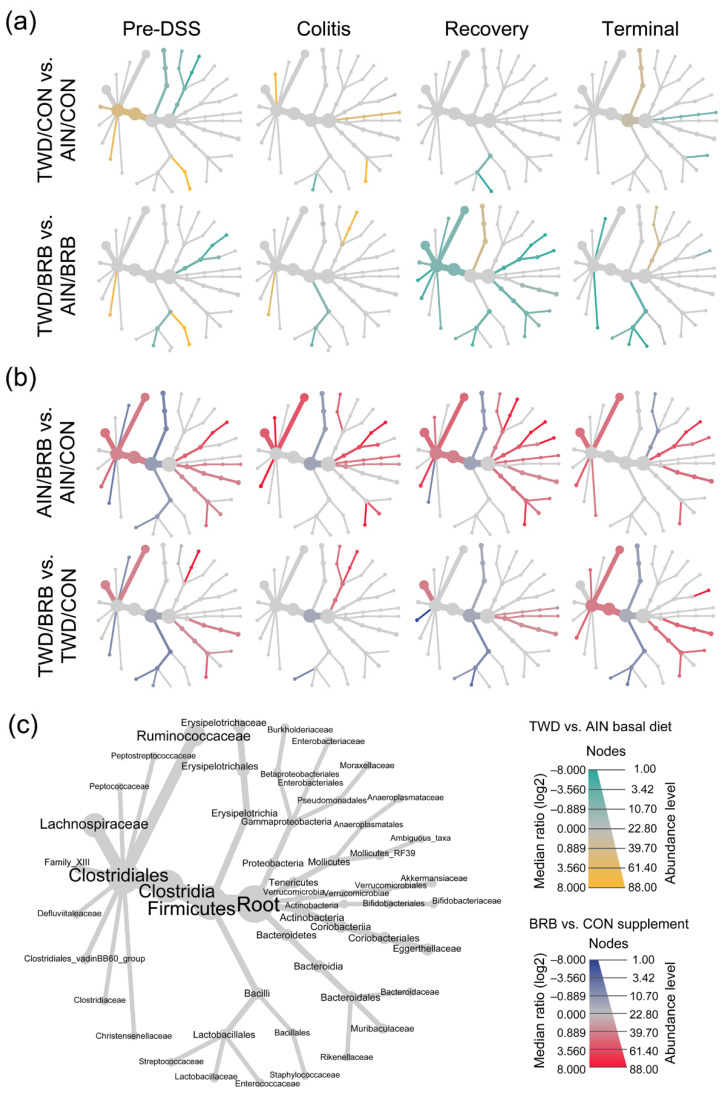
Fecal microbiome community structures depicted as heat trees showing the relative abundance ratios for selected comparisons of basal diet and BRB supplement at each experimental time point (experiment B). (**a**) Comparisons of TWD versus AIN basal diet with or without BRB supplementation (green-to-yellow color bar, with yellow indicating greater abundance in TWD-fed mice; top legend). (**b**) Comparisons of BRB versus CON supplement for AIN or TWD basal diets (blue-to-red color bar, with red indicating greater abundance in BRB-supplemented mice; bottom legend). (**c**) Phylogenetic structure of fecal microbiome bacteria community.

Given the multi-level experimental model incorporating multiple time points, two basal diets and two treatment conditions, our analyses of the fecal microbiome profiles proceeded in a stepwise fashion. First, we considered the overall dynamics of the microbiome in the context of this animal model of inflammation-associated colorectal cancer. Briefly, drastic changes in the composition of the gut microbiome were observed over the course of disease development, from a healthy gut prior to AOM + DSS treatment, during active colitis and progressing through recovery to tumorigenesis, notwithstanding the basal diet or BRB supplement provided (Figure 6, Figure 7, Figure 8, Figures S10 and S11). For example, a decrease in the relative abundance of Akkermansiaceae, specifically *A. muciniphila*, was observed in mice experiencing active colitis, with further loss through recovery to the terminal time point (Figure S10a). The population of Enterobacteriaceae increased during active colitis; then, they were substantially reduced compared to both the colitis and pre-DSS time points with the lowest relative abundance evident at recovery and terminal time points. Bacteroidaceae and Clostridiaceae populations were similarly lower at recovery and terminal time points (Figure S10a). Bifidobacteriaceae (*Bifidobacterium spp*.) were overall very slightly less abundant during colitis (*p* = 0.0258), yet 7% more abundant during the recovery phase before returning to baseline by the terminal time point. Alternatively, bacteria belonging to the Erysipelotrichaceae family were relatively more abundant from the colitis time point onward to the study end. Finally, populations of Lachnospiraceae, Muribaculaceae, Rikenellaceae and Ruminococcaceae were reduced in mice experiencing active colitis, yet appeared to recover shortly thereafter to pre-DSS abundance levels (Figure S10a). Considered collectively, these changes in the microbiome population indicate an overall increase in the shift in the Firmicutes-to-Bacteroidetes ratio favoring firmicutes during active colitis and through recovery with a partial recovery back to the pre-DSS baseline in this mouse model of CAC (Figure S10b).

The remainder of the analyses focused on effects of the basal diet and/or BRB supplement within each experimental time point. The pre-DSS time point revealed the impacts of basal diet on the gut microbiome prior to chemically triggered gut inflammation or carcinogen exposure. Relatively few significant effects of the AIN or TWD basal diets on the microbiome composition were noted (Figure 8a, File S4). Of note, the population of Erysipelotrichaceae was 15% lower in TWD/CON-fed mice compared to AIN/CON (*p* = 0.0237), and that of Clostridiaceae was 15% more abundant in TWD/CON compared to AIN/CON (*p* = 0.0014). During active colitis, the relative abundances of Akkermansiaceae and Lactobacillaceae were 470% greater or 73% lower, respectively, in mice fed the TWD/CON diet compared to AIN controls (*p* = 0.0286 or 0.0048, respectively).

**Figure 9:**
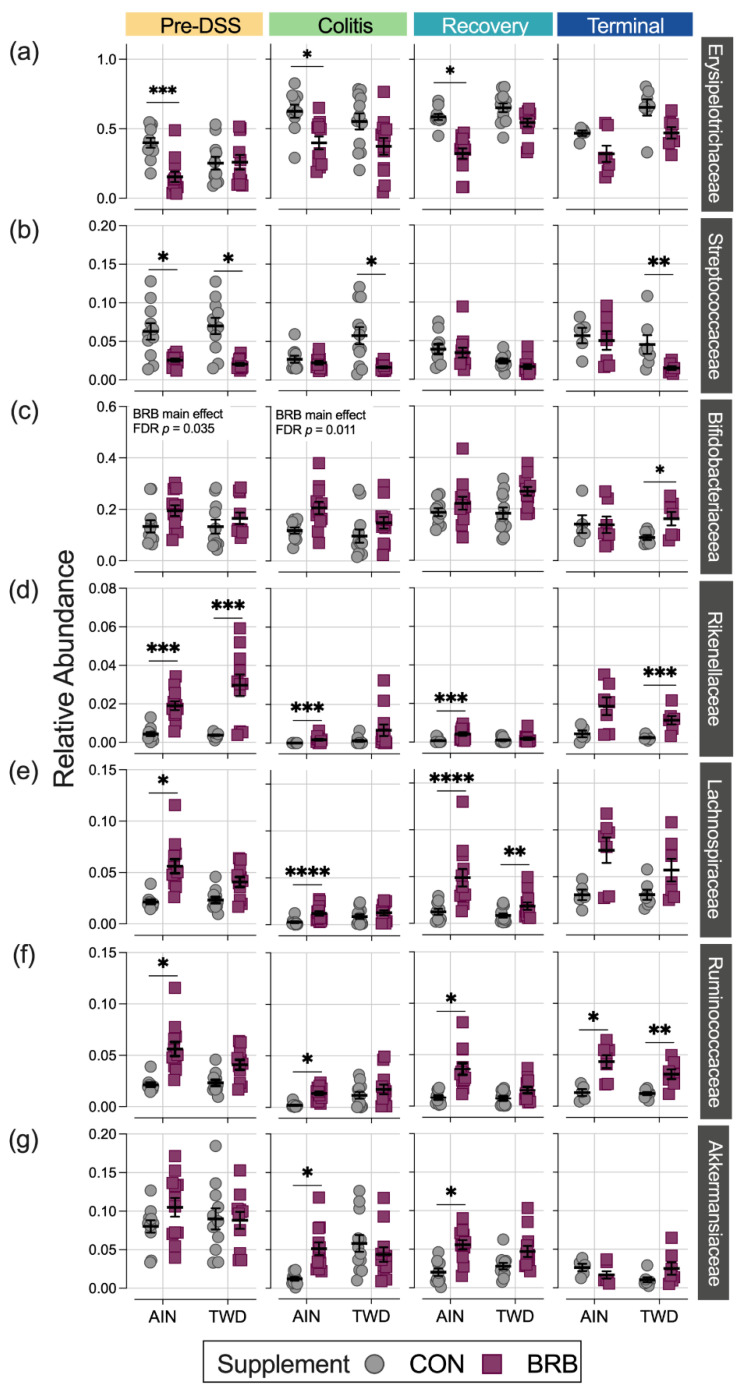
Relative abundance of select bacteria families of interest for each experimental time point (experiment B). (**a**) Erysipelotrichaceae, (**b**) Streptococcaceae, (**c**) Bifidobacteriaceae, (**d**) Lachnospiraceae, (**e**) Rikenellaceae, (**f**) Ruminococcaceae and (**g**) Akkermansiaceae. Data are shown as individual values that represent each cage (as the biological unit) with mean ± SE. For simplified visualization, this plot shows only the statistical results as FDR-corrected *p*-values for comparisons between CON and BRB-supplemented diets as indicated: *, *p* < 0.05; **, *p* < 0.01; ***, *p* < 0.001; and ****, *p* < 0.0001, as outlined in Materials and Methods. Complete results of all metagenomeSeq statistical analyses, including pairwise comparisons by basal diet and across time points, are provided in File S4.

Consumption of BRB had profound effects on the fecal microbiome composition throughout the study, with apparent shifts in bacteria populations dependent on the basal diet at some time points (Figure 8 and Figure 9, File S4). Prior to induction of gut inflammation, BRB intake significantly reduced the population of Erysipelotrichaceae (primarily *Dubosiella newyorkensis*, *Erysipelatoclostridum spp.* and *Turicibacter spp*.) from 39% to just 15% of the fecal microbiome (*p* = 0.0004) in mice fed the AIN basal diet, whereas no change was apparent in mice provided the TWD diet (Figure 9b). This pattern was persistent through colitis and recovery time points, with significant reductions in Erysipelotrichaceae in mice fed the AIN diet, whereas the apparent lower abundance in TWD-fed mice was not statistically significant. BRB markedly reduced the relative abundance of Streptococcaceae (primarily *Lactococcus spp*.) in mice provided either the AIN or TWD diet at the pre-DSS time point (*p* = 0.0045 and 0.0006, respectively) (Figure 9b). However, BRB was effective at lowering Streptococcaceae only in TWD-fed mice during active colitis, perhaps because the relative abundance of this taxa was notably lower in AIN-fed mice at this time point. No further effect of BRB on this population was evident during recovery or at the terminal time point.

BRB supplementation appeared to increase the population of Bifidobacteriaceae (*Bifidobacterium spp*.), as a significant main effect of BRB supplementation, irrespective of diet, was observed prior to DSS treatment and through active colitis and in mice fed the TWD diet at the terminal time point (Figure 9c). Similarly, the population of Rikenellaceae (*Alistipes uncultured bacterium*) increased from just 0.43% of the microbiome in CON mice to 1.9% in BRB-supplemented mice consuming the AIN diet and from 0.38% in CON to 3.0% in BRB-supplemented mice provided the TWD diet at the pre-DSS time point (*p* ≤ 0.001) (Figure 9d). Although less pronounced, this increase in Rikenellaceae was evident for AIN-fed mice during active colitis and through recovery, although not statistically significant by the terminal time point. In mice fed TWD, the effect of BRB was less pronounced during colitis and recovery but as significant at the terminal time point, with an increase from 0.26% in CON compared to 1.2% in BRB-supplemented mice (*p* = 0.0008). Prior to onset of colitis, Lachnospiraceae relative abundance was significantly greater in mice provided BRB on the AIN basal diet (10.3%) compared to controls (3.3%), although not in mice provided the TWD basal diet (Figure 9e). This pattern was consistent during active colitis, although by recovery from gut injury, BRB supplementation effectively increased the relative abundance of Lachnospiraceae for mice fed either the AIN or TWD basal diets. By the end of the study, an apparent greater abundance of this taxa persisted, although it was variable among the cages within the BRB supplemented groups and was not statistically significant for either basal diet.

BRB supplementation was also effective at shifting the relative abundance of Ruminococcaceae (including *Ruminoclostridium spp.*, *Oscillibacter* and *Instestimonas*) throughout the complete study, with an increase from 2.1% to 5.6% of the microbiome in CON compared to BRB-fed mice prior to DSS treatment (*p* = 0.0006), a dampened response during colitis (from 0.2% for CON to 1.4% for BRB; *p* < 0.0001), followed by robust increases through recovery (0.8% for CON and 3.6% for BRB; *p* < 0.0001) and the terminal time points (1.3% in CON increased to 4.3% for BRB; *p* = 0.0482) (Figure 9f). Although a similar trend of elevated population of Ruminococcaceae in TWD-fed mice was also apparent, this response did not reach statistical significance until the terminal time point (*p* = 0.0089). Lastly, of the selected taxa for discussion, Akkermansiaceae relative abundance was higher in mice fed the AIN diet with BRB supplementation during active colitis (increase from 1.2% of bacteria population to 5.1% with BRB; *p* < 0.0001) and through recovery (2.0% in CON increased to 5.6% in BRB-fed mice; *p* = 0.0023) (Figure 9g). BRB supplementation did not appear to affect Akkermansiaceae abundance in mice fed TWD at any study time point.

**Figure 10:**
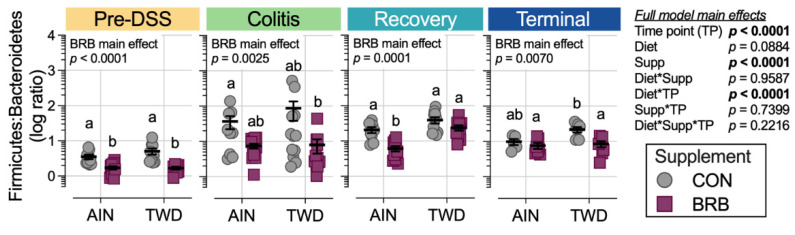
Ratio of Firmicutes to Bacteroidetes for each experimental time point (experiment B). Ratios were determined using normalized count data for each phylum. Data are shown as individual values representing each cage (as the biological unit) with mean ± SE. The table shows the statistical model main effects, including all experimental factors, and different letters indicate that experimental groups are significantly different (*p* < 0.05), as determined by statistical methods outlined in the Materials and Methods section.

At the phylum level, these shifts in the microbiome over the course of disease development led to substantial alterations in the ratio of Firmicutes to Bacteroidetes (F:B), with significant main effect of BRB supplementation (*p* = 0.0001) but with a trend of effect of basal diet (*p* = 0.084) (Figure 10). The F:B ratio was overall lower prior to DSS treatment, with a significant decrease from 3.5 in CON mice to 1.7 for BRB-supplemented mice fed the AIN diet (*p* = 0.0021). Similarly, for mice provided TWD, the ratio decreased from 6.4 to 4.2 with addition of BRB (*p* = 0.0013). During colitis, the comparisons between BRB-supplemented and CON diets were not statistically significant for either AIN or TWD basal diets, although an overall main effect of BRB was evident (*p* = 0.0025). During recovery from DSS-induced gut injury, BRB was effective at reducing the F:B ratio from 20.7 to 6.1 in mice provided the AIN basal diet (*p* = 0.0007) but not in those provided TWD (*p* = 0.3382). By the end of the study, however, this pattern was reversed, with BRB effectively decreasing the F:B ratio from 21.6 to 8.5 in mice fed the TWD (*p* = 0.0115) but not the AIN diet (*p* = 0.8144).

#### 3.2.5. Alpha and Beta Diversity of Fecal Microbiome (Experiment B)

**Figure 11:**
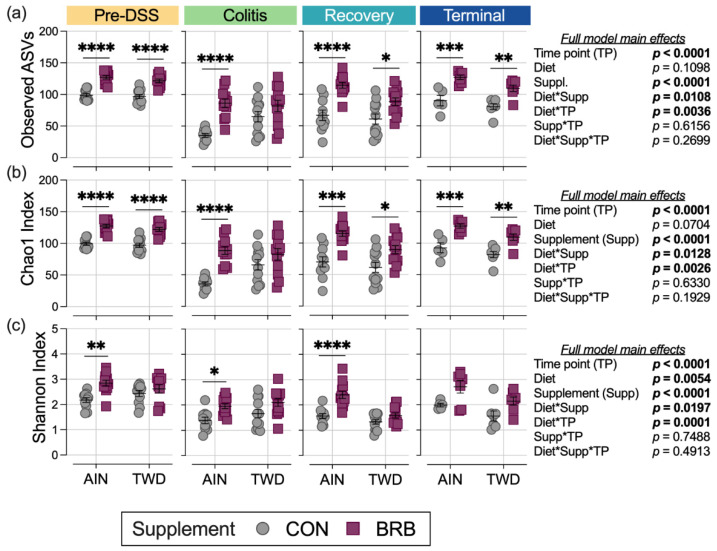
Alpha diversity of mouse fecal microbiomes for each experimental time point (experiment B). Alpha diversity measures include (**a**) observed ASVs, (**b**) the Chao1 index and (**c**) the Shannon index. Data are shown as individual values representing each cage (as the biological unit) with mean ± SE. Inset tables show the statistical model main effects, including all experimental factors, for each α-diversity measure. For simplified visualization, this plot shows only the statistical results for comparisons between CON and BRB-supplemented diets, as indicated: *, *p* < 0.05; **, *p* < 0.01; ***, *p* < 0.001; and ****, *p* < 0.0001, as outlined in Materials and Methods. Complete results of these statistical analyses, including comparisons within and across time points, are provided in Tables S2–S4.

Alpha diversity was determined using three measures: observed ASVs, Chao1 index and Shannon index. First considering the microbiome composition over the course of disease development, irrespective of basal diet or BRB supplementation, α-diversity was substantially reduced in mice experiencing colitis compared to pre-DSS baseline with prolonged loss of taxa through recovery (*p* < 0.0001 for all α-diversity measures; Figure 11, Table S2). By the end of the study, α-diversity measures were more similar to the pre-DSS baseline (*p* > 0.05 for observed ASVs and Chao1 index), although when considered as a weighted measure by the Shannon index, the overall α-diversity was still significantly reduced (*p* = 0.0008; Table S2).

The main model analyses revealed profound effects of BRB treatment on α-diversity throughout the study, with mostly consistent effects in mice provided either the AIN or TWD basal diets for observed ASVs and the Chao1 index (Figure 11a,b; Table S3). One notable exception was noted during active colitis, when BRB supplementation appeared less effective in improving α-diversity in mice fed TWD. Furthermore, this pattern of reduced BRB efficacy in mice fed the TWD basal diet was further apparent for the Shannon index at the pre-DSS baseline, during colitis and through recovery (Figure 11c).

Beta diversity was determined using both weighted and unweighted unifrac distances to explore the microbiome community profiles based on relative abundance and rare taxa, respectively. As anticipated, for unweighted unifrac distance β-diversity (Figure S12a), clear separations of microbiome samples were evident at each experimental time point with the most distinct profiles apparent in mice experiencing active colitis through the recovery period 14 days later (permanova *p* < 0.001 for each diet/supplement group). Interestingly, when considering the composition of the microbiome weighted for abundance, the pre-DSS baseline microbiome appeared most distinct (Figure S12b) and still notably different from microbiomes at the terminal time point, suggesting that recovery from gut injury led to long-term changes in fecal microbiome profiles (permanova *p* < 0.001 for each diet/supplement group).

**Figure 12:**
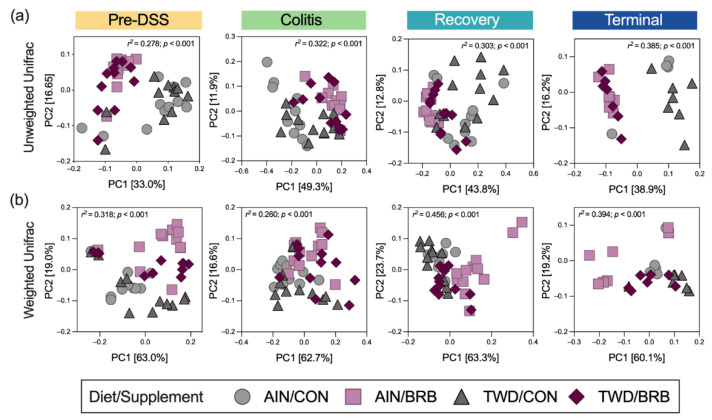
Beta diversity of mouse fecal microbiomes for each experimental time point (experiment B). Principal coordinate plots depicting fecal microbiome beta diversity using (**a**) unweighted or (**b**) weighted unifrac distances are shown using the first two components. The variations attributed to PC1 and PC2 are shown along with the *r*^2^ and permanova *p*-values for each plot.

Dietary supplementation with BRB caused marked shifts in the composition of the fecal microbiome, as assessed using either weighted or unweighted unifrac distance measures (Figure 12), whereas basal diet appeared to have only modest or no apparent effects on bacteria profiles at any of the disease stages in this experiment. Following two weeks of experimental treatments at the pre-DSS baseline, BRB appeared to shift the microbiome composition, such that the profiles were clearly distinct compared to their CON counterparts. This pattern was mostly consistent at the colitis time point (permanova *p* < 0.001), although more overlap among experimental groups was apparent for weighted unifrac β-diversity. By the recovery time point, the fecal microbiomes of TWD/BRB mice appeared less distinct from those provided the CON diet for either weighted or unweighted β-diversity, although AIN/BRB microbiomes were still distant. At the study end, unweighted β-diversity revealed persistent distinct microbiomes for mice supplemented with BRB fed either diet, whereas the weighted measure suggested only a strong segregation of microbiomes from AIN/BRB mice (Figure 12), suggesting that the long-term effects of BRB supplementation may be more profound for rarer taxa, which more heavily influenced the unweighted unifrac analysis.

#### 3.2.6. Functional Metagenomics and Longitudinal Analyses (Experiment B)

**Figure 13:**
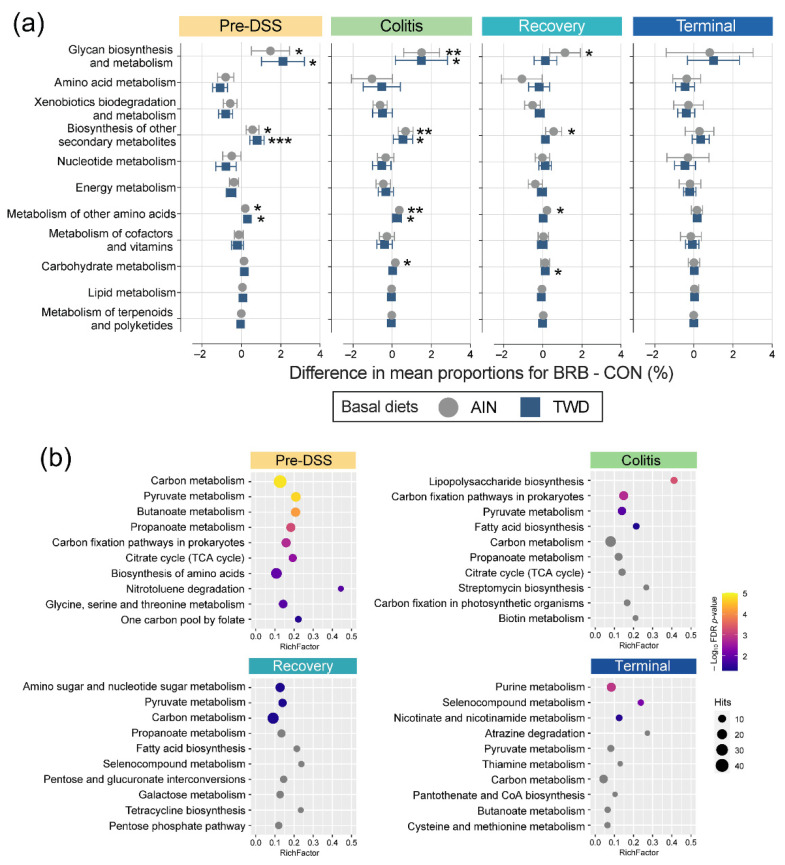
Metagenome predicted functions classified using KEGG metabolism orthology with tax4fun (experiment B). (**a**) Differences in mean proportions for BRB-supplemented and CON treatments for mice fed either AIN or TWD basal diets. Values are the differences between proportions with 95% confidence intervals. *, *p* < 0.05; **, *p* < 0.01; and ***, *p* < 0.001, as determined by Fisher’s exact test followed by Benjamini–Hochberg method to adjust for multiple comparisons for the full data set. Proportions for each diet and supplement combination at each time point are provided in Figure S13b. (**b**) Pathway enrichment of significant KEGG level 2 terms associated with the fecal microbiome of BRB-fed mice (AIN- and TWD-fed combined). Values shown are the term count (number of terms associated with the metabolism pathway), the enrichment factor (number of significant terms/total terms in pathway) and the FDR-corrected *p*-value.

Functional potential prediction revealed significant differences in the representation of KEGG orthology terms when comparing mice supplemented with BRB and the controls fed either the AIN or TWD basal diets (Figure 13a and Figure S13). At the pre-DSS baseline, the KEGG metabolism level 1 terms of glycan biosynthesis and metabolism, biosynthesis of other secondary metabolites and metabolism of other amino acids were significantly enriched in BRB- supplemented mice compared to CON counterparts for both basal diets. This pattern was consistent during active colitis, with one additional category, carbohydrate metabolism, slightly but significantly enriched in AIN/BRB mice only. During recovery, this effect of BRB was apparent only for AIN-fed mice, with the exception at this time point of carbohydrate metabolism being very slightly enriched for TWD-fed mice. By the study end, no significant enrichments in level 1 KEGG terms were noted.

The lists of significant terms identified via metagenomeSeq analyses were subject to pathway association analysis to identify enriched level 2 pathways for BRB supplementation, irrespective of basal diet, at each time point (Figure 13b). Addition of BRB to the mouse diet appeared to strongly shift the functional capacity of the gut microbiome in favor of carbon metabolism, pyruvate metabolism, butanoate metabolism and propanoate metabolism, along with several other pathways. Of note, only during active colitis, a very strong enrichment in taxa associated with lipopolysaccharide biosynthesis was noted, as well as shifts favoring carbon fixation pathways in prokaryotes, pyruvate metabolism and fatty acid biosynthesis. Like the colitis time point, relatively few enriched functional pathways were identified for BRB-supplemented gut microbiomes during recovery from gut injury, with amino sugar and nucleotide sugar metabolism uniquely enriched along with pyruvate metabolism and carbon metabolism, as in prior stages. By the end of the experiment, functional analyses revealed a unique pathway set for BRB-supplemented mice, including purine metabolism, selenocompound metabolism, nicotinate and nicotinamide metabolism.

**Figure 14:**
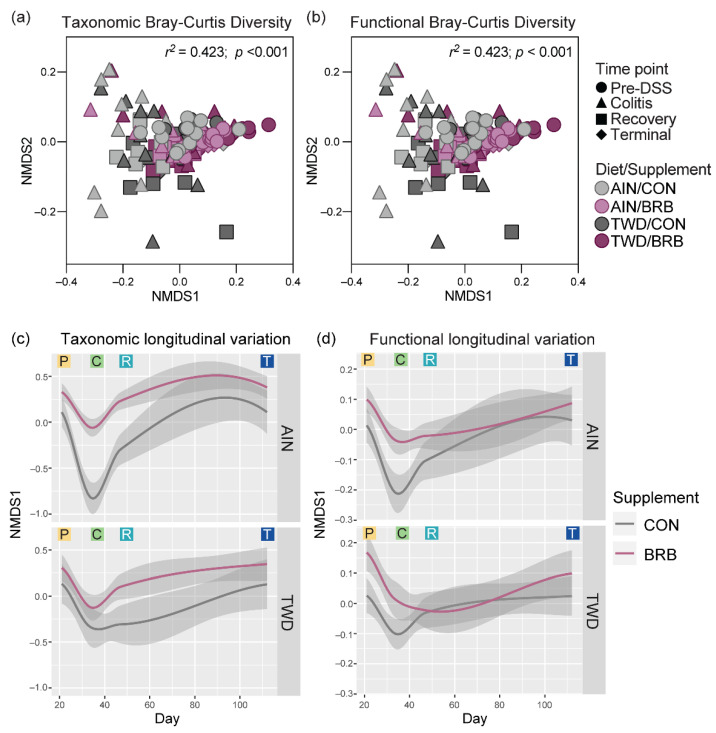
Longitudinal analysis of fecal microbiome taxonomy and functional capacity (experiment B). (**a**,**b**) NMDS ordination plots of the taxonomic (**a**) and functional (**b**) β-diversity for fecal microbiomes of mice fed either CON or BRB-supplemented diet with either AIN or TWD basal diets for all experimental time points. Functional beta diversity was measured as the Bray–Curtis dissimilarity based on KEGG term abundances, while taxonomic beta-diversity values represent the Bray–Curtis dissimilarity based on ASV abundances. (**c**,**d**) Longitudinal variation shown as the first dimension plotted over experimental day for taxonomic (**c**) and functional (**d**) diversity. Loess-smoothed trajectories of microbiomes from each experimental group are plotted; gray shading represents the 95% confidence interval. P, pre-DSS; C, colitis; R, recovery; and T, terminal time points.

To explore longitudinal trends in microbiome taxonomic and functional diversity, Bray–Curtis β-diversity was analyzed using non-metric multi-dimensional scaling (NMDS) with the complete experimental data set across all time points. In this visualization, BRB supplementation appears to be the primary experimental factor driving taxonomic diversity, with time point as a probable secondary factor (Figure 14a). Alternatively, these distinctions are less clear when considering the microbiome functional capacity (Figure 14b). The longitudinal variation of samples along the first NMDS dimension shows how microbiome populations diverge with respect to taxonomy proximal to onset of colitis (circa day 33) with continued divergence persisting through recovery (circa day 45) and then largely resolving by the end of the experiment (Figure S14 and Figure 14c,d). Of note, a clear divergence in both taxonomic and functional variation is evident during active colitis for mice fed TWD as compared to the AIN diet without BRB supplementation (Figure S14); yet, this divergence largely resolves through recovery and on to the tumorigenesis phase in this disease model. Furthermore, a strong taxonomic divergence was associated with response to BRB supplementation, although this appears more pronounced for mice fed the AIN diet compared to those provided TWD (Figure 14c). Longitudinal analysis of the gut microbiome functional capacity revealed similar divergence in microbiomes proximal to colitis when fed either CON or BRB-supplemented diets, although resolution appears to be more rapid around the recovery time point (Figure 14d).

#### 3.2.7. Fecal Short-Chain Fatty Acid Content (Experiment B)

**Figure 15:**
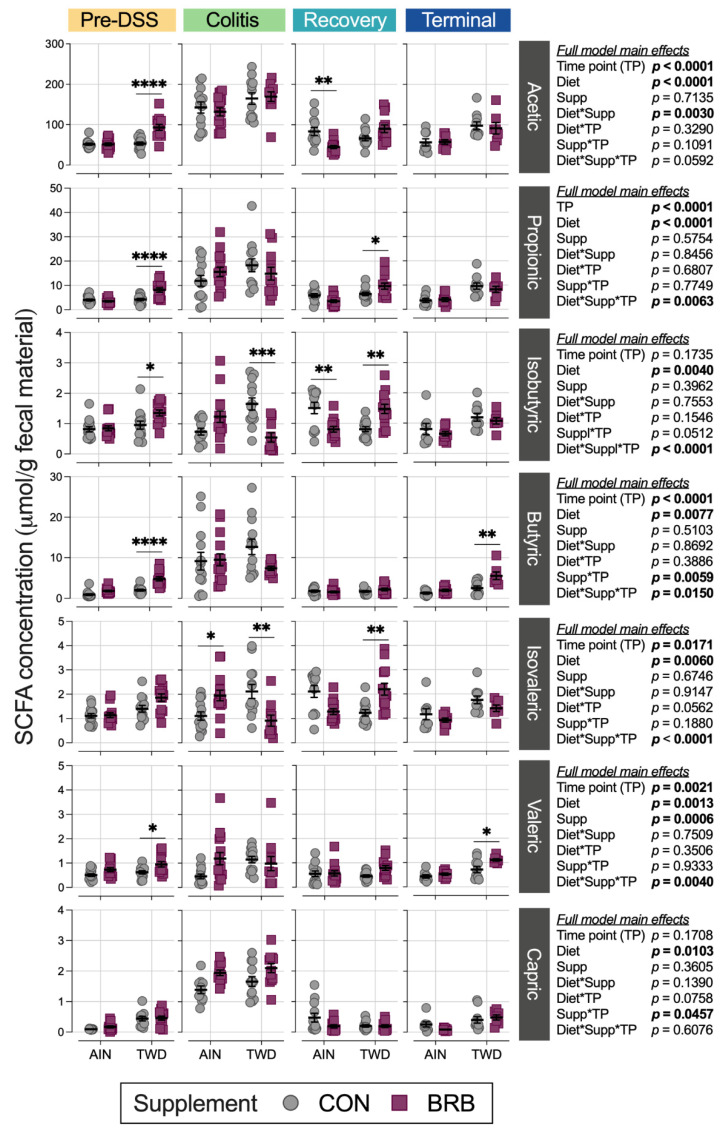
Short-chain fatty acid concentrations in fecal samples from mice fed CON or BRB-supplemented diets at each experimental time point (experiment B). Data are shown as individual values that represent each cage (as the biological unit) with mean ± SE. For simplified visualization, this plot shows only the statistical results as FDR-corrected *p*-values for comparisons between CON and BRB-supplemented diets, as indicated: *, *p* < 0.05; **, *p* < 0.01; ***, *p* < 0.001; and ****, *p* < 0.0001, as outlined in Materials and Methods. Complete results of all statistical analyses, including pairwise comparisons by basal diet and across time points, are provided in Tables S5–S7.

Mice provided BRB in the context of the TWD basal diet produced significantly higher amounts of acetic (75% ↑, *p* < 0.0001), propionic (93% ↑, *p* < 0.0001), isobutyric (42% ↑, *p* = 0.0389), butyric (240% ↑, *p* < 0.0001) and valeric (52% ↑, *p* = 0.0382) acids compared to their AIN-fed counterparts during the initial phase of the study prior to DSS treatment (Figure 15, Tables S5–S7). Notably, this observation correlates closely with the higher total food and energy intakes in mice provided the TWD/BRB diet in the first three weeks of the experiment (Figure S7). During active colitis, the overall production of acetic, butyric, propionic and valeric acids was significantly elevated (Table S5), although BRB supplementation did not significantly alter these specific SCFAs for mice fed either basal diet (Figure 15). Rather, isobutryic and isovaleric acids were significantly reduced by 67% (*p* = 0.0005) and 57% (*p* = 0.0060), respectively, in mice provided the TWD/BRB experimental diet compared to TWD/CON, while isovaleric acid was significantly elevated by 76% (*p* = 0.0457) in mice provided the AIN/BRB diet compared to AIN/CON. As the animals recovered from gut injury, BRB supplementation significantly reduced acetic acid (46% ↓, *p* = 0.0047) and isobutyric acid (47% ↓, *p* = 0.0032) in fecal samples from AIN-fed mice, whereas BRB elevated propionic (50% ↑, *p* = 0.0467), isobutryic (82% ↑, *p* = 0.0043) and isovaleric (79% ↑, *p* = 0.0040) acids in mice provided the TWD basal diet. By the study end, overall SCFA in fecal samples returned to the pre-DSS baseline (Table S5). In the long term, compared to control mice, BRB supplementation increased butyric acid fecal concentration by about 2-fold (*p* = 0.0021) and valeric acid by 55% (*p* = 0.0209) in mice provided the TWD but not in their AIN-fed counterparts.

## 4. Discussion

Consumption of a rodent diet that emulates the typical American pattern with respect to macro- and micronutrient profiles has been repeatedly shown to enhance symptoms of colitis, inflammatory cell infiltration, mucosal injury and tumor development in murine models of colitis-associated colorectal cancer [12,35]. Herein, we report the findings from the first investigation to explore the purported benefit of dietary supplementation with BRB for gut health and modulation of the gut microbiome in a mouse model of Western-diet enhanced colitis and colitis-associated colorectal cancer. The results of a pilot study suggested that BRB supplementation ameliorated colitis symptoms and reduced colon tumorigenesis in mice provided the TWD. Moreover, assessment of the fecal microbiome suggested that BRB consumption led to shifts in the gut microbiome in favor of health-promoting species. However, in a more extensive follow-up study, the suppressive effects of BRB supplementation on colitis and CAC were mixed, with no apparent effects on colitis symptoms, colon inflammation or mucosa injury, yet an apparent improvement in colon length and reduced tumor multiplicity by the end of the experiment, irrespective of the basal diet provided. As observed in the pilot study, in the second experiment, BRB supplementation had profound effects on the composition of the fecal microbiome evident even before the onset of colitis, as evidenced by increased alpha diversity and distinct microbiome profiles throughout the study period, during colitis, recovery, and finally, tumorigenesis. Of interest, when considering changes in abundance of individual taxa, we observed more frequent significant changes in their relative abundance values in mice provided the AIN basal diet as compared to those fed TWD.

An unexpected finding in both experiments was the apparent increased energy intake for mice provided the TWD supplemented with BRB. In prior investigations employing the TWD, we observed that mice fed TWD typically consume less food, such that their overall energy intake is consistent with that of mice provided the AIN diet [12,13,35,46]. However, TWD/CON food intake for experiment B of this study was statistically greater compared to their AIN/CON counterparts, leading to elevated energy intake. In both experiments of this investigation, addition of BRB to the TWD basal diet led to further increased intake of food, higher energy intake and, consequently, increased body weight and fat mass. However, these effects were not apparent for mice provided the AIN basal diet, pointing to an interaction between the TWD nutritional profile—with its higher energy density and unbalanced micronutrient composition—and dietary supplementation with BRB. Importantly, CON and BRB-supplemented diets were matched for total sugar and fat content. Thus, it is reasonable to surmise that the addition of BRB increased the palatability of the rodent diet, presumably due to the differing sugar profile of black raspberries (fructose and glucose) compared to the semi-purified diets containing only sucrose. Future studies should explore the potential adverse effects of the TWD on the gut–brain axis and neuroendocrine control of hunger and satiety—effects, which may explain why hyperphagia was notably pronounced in mice provided with the more palatable BRB diet.

As previously observed in multiple studies, mice consuming TWD experienced more severe colitis symptoms, increased inflammation and mucosal damage in the colon epithelium and elevated tumor development compared to mice consuming the standard AIN [12,35], a pattern that persisted in the current studies. Given the link between consumption of this Western diet by rodents and evident promotion of colitis and CAC, it is important to identify the functional foods abundant in bioactive chemicals with potent anti-inflammatory, antioxidant and/or anti-cancer properties. Herein, we report that BRB supplementation consistently reduced tumor multiplicity in a mouse model of Western-diet enhanced CAC. However, the findings for symptoms of colitis and colon inflammation were equivocal. Several previous studies have explored the effects of black-raspberry-supplemented diets in both murine cancer and inflammatory disease models, although not in the context of a Western dietary pattern. Apc*^Min^*^/*+*^ mice fed AIN76A supplemented with 5% whole freeze-dried BRB powder had a decrease in polyp burden and size in the small intestine and colon compared to control diet [47]. Dong et al. compared different doses of protocatechuic acid (PCA) and 5% BRB supplemented in a AIN76A diet administered to Apc*^Min^*^/*+*^ mice, resulting in a greater effect of 5% BRB on polyp number and size, tumor incidence and shift of pro-inflammatory bacterial species to anti-inflammatory promoting taxa [48]. Using a similar experimental model of CAC in C57BL6/J mice, Chen et al. observed decreased colon tumor incidence and multiplicity for mice provided chow supplemented with 5 or 10% BRB [25]. When added to the AIN76 diet, black raspberries, their anthocyanin bioactives and a microbial metabolite of anthocyanins have also been shown to be effective at suppressing chemical carcinogenesis in a rat model of esophageal cancer [15,22].

In addition to its apparent anti-cancer properties, evidence also points to the anti-inflammatory effects of dietary supplementation with BRB in animal studies. Using a DSS-induced model of UC, Montrose et al. reported that addition of BRB to the AIN76 basal diet suppressed key pro-inflammatory cytokines, including tumor necrosis factor-alpha (TNFα) and interleukin 1-beta (IL1β), thus inhibiting cyclooxygenase 2 (COX2) [23]. Wister rats fed a high-fat diet supplemented with 2.5% or 5% BRB freeze-dried powder for 8 weeks had decreased levels of IL1β, IL6 and COX2 [49]. Multiple studies using the *IL10* knockout mouse model for UC showed that supplementing the AIN76A diet with 5% BRB led to decreased inflammatory cell infiltration in colon tissues, which the authors concluded was due to the BRB supplement correcting dysregulated TLR4 in colonocytes, decreasing prostaglandin E2 and inhibiting aberrant epigenetic pathways by decreasing β-catenin translocation levels [50,51]. Additionally, BRB decreased NF-κB p65 expression, which reduced aberrant DNA methylation of tumor suppression genes in the Wnt pathway in a C57BL/6J mouse model of UC using the AIN76A diet supplemented with 5% BRB powder [52]. Collectively, this evidence suggests that supplementation with BRB in mice consuming a standard diet decreased inflammatory cytokines, ameliorated TLR4 dysregulation and reduced methylation of tumor suppression genes.

A key finding of this investigation was that consumption of BRB markedly altered the composition of the fecal microbiome in a dynamic way over the course of disease development and, ultimately, tumorigenesis. Anthocyanin-rich BRB promoted growth of more diverse microbial communities in the colon, which were notably distinct from time-matched controls, as evidenced by elevated α-diversities and highly divergent β-diversities (experiment B). Comparable to these findings, Gu et al. reported that dietary supplementation of AIN93G with 10% freeze-dried BRB powder diet in healthy male mice increased richness of the mucosal and luminal microbiomes of the colon while also promoting growth of distinct microbiota populations [17]. In a UC model of C57BL/6J mice provided 1% DSS in water for two weeks, the AIN76A diet supplemented with 10% BRB powder had an attenuating effect on microbial richness compared to control diet and higher diversity than mice fed only the control diet [53]. Collectively, BRB supplementation promotes anti-inflammatory and anti-cancer properties while also enriching the gut microbiome.

Shifts in relative abundance in major phylum favoring Firmicutes compared to Bacteroidetes (F:B) has been previously linked to chronic inflammatory disease, including obesity and inflammatory bowel disease (IBD) [54,55]. As noted for the more extensive experiment B, the F:B ratio was consistently reduced in BRB-supplemented diets prior to onset of colitis, during active colitis, through recovery and colon tumorigenesis. Driving this decrease in the F:B ratio was a lower relative abundance of Erysipelotrichaceae and Streptococcaceae in BRB-fed mice compared to CON diet. Of note, members of these bacteria families have been previously found in high abundance in patients diagnosed with UC or Crohn’s disease [56,57]. Schaubeck et al. observed a significant increase in Erysipelotrichaceae in mice that developed tumor necrosis factor (TNF)-driven CD-like transmural inflammation [58]. In this investigation using a mouse model of colitis and CAC, the abundance of Erysipelotrichaceae was higher in mice (experiment B) during active colitis and remained elevated through recovery and colon tumor development. Alternatively, an increased abundance in this bacteria family was associated with a 12% lower risk of IBD and 14% reduced risk of UC in humans [59]. Insulin-resistant mice fed a high-fat, high-sugar diet supplemented with cyanidin-3-glucoside (C3G) extract, commonly found in blueberries, reduced Erysipelotrichaceae and Streptococcaceae relative abundance [60], a finding similar to this study in which BRB supplementation reduced both Erysipelotrichaceae and Streptococcaceae prior to DSS-induced gut injury and during active colitis, with different outcomes depending on the basal diet provided.

Bacteria belonging to the Bifidobacteriaceae family (*Bifidobacterium pseudolongum* in experiment A and unidentified species in experiment B) were enriched with BRB supplementation in this investigation, most notably during active colitis in both experiments. Bifidobacteriaceae dominate the gut microbiome of infants but decrease in abundance by the time a child reaches three years of age [61]. Species belonging to the Bifidobacteriaceae family suppress the immune response, increase acetate production and improve intestinal barrier function in infants and provide protective effects in CD patients [62]. In a recent study that explored colon mucosa and tumor-associated microbiomes by comparing age-matched wild-type F344 rats and Apc-mutated Pirc rats, Vitali et al. reported that the colon tumor tissue-associated microbiome was enriched for Bifidobacteriaceae, suggesting a relationship between bacteria in this family and the tumor environment [63]. Previous studies exploring the effects of dietary supplementation with BRB in animal models of IBD or CAC did not report changes in abundance of Bifidobacteriaceae taxa, contrary to our findings [17,53].

In the current study, BRB supplementation increased the relative abundance of Lachnospiraceae, Ruminococcaceae and Rikenellaceae prior to, during and after carcinogen exposure. Members of the Lachnospiraceae family are butyrate-producing and degrades cellulose and hemicellulose derivatives of plant fibers, leading to increased bioavailability for host absorption and contributing to intestinal homeostasis [64]. In a study of 42 CRC patients and 89 matched controls, Sinha and colleagues determined a significant association of CRC diagnosis with reduced abundance of Lachnospiraceae in feces [65]. In addition, the relative abundance of Lachnospiraceae decreased with increasing severity of bowel inflammation in a study of Chinese patients with UC [66]. Multiple studies have shown that dietary supplementation with anthocyanin-rich foods alters Lachnospiraceae abundance in the gut with purported health benefits. For example, addition of an anthocyanin-rich extract from bilberries to the diet promoted the growth of Lachnospiraceae in aged rats [67]. Additionally, Chen et al. reported that treatment with the anthocyanin cyanidin-3-O-glucoside ameliorated chemically induced gut dysbiosis, including increased abundance of Lachnospiraceae_NK4A136_group, and protected against intestinal mucosa damage [68].

In this investigation, we observed different responses to BRB intervention for taxa in the Ruminococcaceae family, with apparent reduced abundance during active colitis in the pilot experiment and clearly elevated abundance in the more extensive study, most notably in mice fed the AIN diet prior to gut injury, during active colitis, through recovery and, for both basal diets, on to tumor development. Ruminococcaceae are butyrate-producing bacteria necessary for intestinal barrier functions, and a decrease in the relative abundance of Ruminococcaceae has been shown to promote secondary bile deficiencies and intestinal inflammation [69,70,71]. Interestingly, others have also reported mixed outcomes for Ruminococcaceae abundance following intervention with anthocyanin-rich foods. For example, Pan et al. reported that healthy F344 rats fed the AIN76G diet with 5% BRB had increased Ruminococcaceae in feces [24]. Alternatively, dietary supplementation with 5% BRB or protocatechuic acid, a microbial-derived metabolite of black raspberries, apparently decreased the relative abundance of fecal *Ruminococcus gnavus* in the APC*^Min^*^/*+*^ mouse model of small intestinal cancer [48]. Furthermore, in C57BL/6J mice fed a high-fat basal diet, blueberry and cranberry anthocyanin extracts were shown to reduce the relative abundance of Ruminococcaceae in the fecal microbiome as compared to high-fat control mice [72]. Finally, Kennedy and colleagues reported that the relative abundance of Rikenellaceae is reduced in CD patients compared to healthy controls [73]. Anthocyanin extract from blueberries and cranberries added to a high-fat diet reduced the abundance of Rikenellaceae in C57BL/6J mice [72], while blue honeysuckle berries, containing mainly cyanidin 3-glucoside, added to a high-fat diet led to increased abundance of Rikenellaceae in the fecal microbiome of male mice [74].

Within the Verrucomicrobiaceae family, *A. muciniphila* is one of the most widely studied bacterium due to its apparent critical role in sustaining gut homeostasis via promotion of mucin secretion and maintenance of the mucosal layer. Thus, application of *A. muciniphila* as a pro-biotic or identification of diets that promote its abundance are of high interest to leverage its potential for sustaining gut health and supporting the intestinal immune response [75]. However, the relationship between *A. muciniphila* and intestinal inflammatory diseases is unclear (reviewed in Ref [76]), as multiple studies have reported equivocal findings in humans [77,78,79,80,81] and in animal models [82,83,84]. In this investigation, we observed a marked decrease in the abundance of Verrucomicrobiaceae (*Akkermansia* unclassified species) following DSS-induced injury to the gut epithelium, although less so during colitis for TWD-fed mice, suggesting that the highly inflamed and damaged tissue environment was not supportive for this bacterium. Furthermore, we reported that Akkermansiaceae was elevated in mice fed the AIN diet supplemented with BRB during active colitis and through recovery compared to controls, although not in TWD-fed mice (experiment B). Polyphenols derived from Concord grapes have been shown to increase Akkermansiaceae abundance, resulting in enhanced intestinal barrier function and suppression of inflammatory cells in mice fed a high-fat diet [85]. Additionally, multiple studies have shown that addition of either blueberry or cranberry powder or extract to a high-fat, high-sugar diet increased the abundance of *A. muciniphila* after 6 to 11 weeks [72,86]. In this study, we identified the key microbial taxa associated with gut inflammation as possible biomarkers of disease. While this study included a dynamic assessment of the microbiome, it remains unclear whether changes in the abundance of bacteria, such as *A. muciniphila*, are indeed causative of altered colitis status or are a consequence of changes in gut inflammation status.

In a prior longitudinal study of the gut microbiome in a well-established mouse model of IBD, Sharpton et al. suggested that the taxonomic structure and functional capabilities of the gut microbiome shifted through disease development, and these changes correlated with immune activation [87]. In the current study, we observed transient, diet-dependent divergence of microbial taxonomic and functional diversity, most evident in the healthy mice before DSS-induced gut injury and during active colitis, which then resolved—especially with respect to functional diversity—during recovery from gut injury. This divergence was apparent for mice consuming both basal diets, although clearly more pronounced in mice fed the AIN. Prior to gut insult, our metagenomic analysis of BRB-fed mice compared to controls revealed differences in KEGG level 1 terms, including increased glycan biosynthesis and metabolism, biosynthesis of other secondary metabolites and metabolism of other amino acids. Moreover, enriched KEGG level 2 terms in mice provided BRB included pyruvate, butanoate and propanoate metabolism pathways, notably prior to gut injury. During active colitis and recovery of gut injury, lipopolysaccharide biosynthesis, carbon fixation and fatty acid metabolism pathways were enriched, likely a consequence of DSS-induced inflammation and disease progression.

Black raspberry polyphenols, such as phenolic acids, flavanols and anthocyanins [17], are broken down to secondary metabolites, such as SCFAs. For example, the black raspberry freeze-dried powder in this study had a 3.72% *w*/*w* of anthocyanin with cyanidin-3-*O*-glucoside being the dominant structure. The sugar moiety is removed from the cyanidin via β-glucosidase, which enhances glycan metabolism into butyrate [88]. Glucose is actively converted to pyruvate promoting the citric acid cycle, consequently stimulating metabolism of other amino acids. Further, cyanidin is hydrolyzed and cleaved into two important secondary metabolites, protocatechuic acid and pholoroglucinaldehyde [89]. Given the apparent shift in functional diversity of the gut microbiome in mice fed BRB-supplemented diets, we also determined the concentrations of SCFAs in fecal samples. Interestingly, the apparent change in the functional diversity of the gut microbiome associated with BRB supplementation, as indicated by enriched KEGG level 2 terms for pyruvate, butanoate and propanoate metabolism, was associated with shifts in fecal SCFA levels, specifically acetic, propionic and butyric acids prior to gut injury, especially in mice fed the TWD. However, it should also be noted that mice fed the TWD + BRB diet had higher overall energy intake compared to the other experimental groups, which could explain the higher concentration of SCFAs in the feces of these mice. Tu et al. reported that C57BL/6J mice fed the AIN76A diet with 10% BRB had similar increases in butyrate as compared to controls [90]. Butyrate is the least abundant SCFA and is the main energy source for colonocytes. Further, butyrate modulates the immune responses, intestinal barrier function, inhibits histone acetylation and has been shown to have anti-cancer properties (reviewed in Ref [91]).

This study has some limitations that should be considered. First among these are the apparent contradictory findings between the pilot study (experiment A) and the more extensive second study (experiment B), most notably for colitis disease activity index, the histopathology assessment of inflammation and mucosal injury, and tumor burden, although BRB supplementation similarly reduced tumor multiplicity in both studies. Additionally, different results were apparent for the relative abundance of specific microbiome taxa. With respect to microbiome analyses, the two key considerations are (1) the different sequencing technologies, with the methods for experiment B being more robust and achieving much improved sequencing depth to capture more rare taxa; and (2) fewer cages employed in the pilot study limited the statistical power. Given the different sequencing platforms and databases used for mapping sequences to bacteria, comparisons across the two experiments for specific bacteria taxa should be made with caution. Additionally, fecal microbiome profiling determined the relative abundance of various taxa present rather than their absolute abundance; it is possible that apparent changes in the relative abundance of one species may not be due to changes in its actual population size but rather due to the growth or loss of other species in the community. The experimental model of CAC was the same for both studies, yielding the anticipated phenotype outcomes for colitis, histopathology and tumorigenesis for mice fed either the AIN or TWD, as has been observed in several prior studies by our group. Additionally, the diet formulations were consistent across the two experiments, as we used the same source of BRB freeze-dried powder with a consistent anthocyanin profile; thus, the experimental diets were not likely the driving factor in apparent differences in inflammatory response. At this point, we cannot posit a clear explanation for the apparent differences in inflammatory response evident in these experiments. However, we note that repeated pre-clinical experiments with dietary interventions for cancer prevention are rarely provided in the literature, and these mixed results give further weight to the need to consider the reproducibility of pre-clinical studies before advancing such work to clinical settings. Furthermore, the concentration of BRB in the mouse diets employed in this study would deliver a dose of anthocyanins (estimated 448 mg/kg per day with estimated human equivalent dose of 7.6 mg/kg based on surface area allometric scaling) approximately 5-fold greater than could reasonably be achieved in humans consuming black raspberries as part of a regular diet (approximately 460 mg anthocyanins in 100 g serving of fresh black raspberries, for a dose of 7.6 mg/kg). Moreover, these berries are a seasonal specialty crop and not widely available in local groceries. However, concentrated anthocyanin-rich extracts and freeze-dried fruit powders (as used in this study) are commercially available all year round.

## 5. Conclusions

In conclusion, dietary supplementation with freeze-dried black raspberry powder appeared to suppress colon tumorigenesis, as evidenced by reduced colon tumor multiplicity in multiple experiments, although the effects on colitis symptoms, colonic inflammation and mucosal damage were inconsistent. Moreover, addition of BRB to the mouse diet shifted the fecal microbiome composition in favor of health-promoting taxa, increased alpha diversity and promoted growth of distinct microbiome populations compared to mice fed the control diets. Importantly, a significant interaction between basal diet and BRB supplement was evident for many experimental parameters, such as body weight and fat mass gain, alpha diversity and relative abundance of several bacteria families, pointing to the importance of the underlying nutritional status of the target population for intervention with functional foods. The key issues still left to be addressed in future investigations include the potential benefit of other anthocyanin-rich foods with differing anthocyanin profiles in this animal model of Western-diet enhanced CAC, the impact of varying anthocyanin-rich food intake patterns to emulate typical human diet (e.g., varied food items, varied intake schedule) and the duration of microbiome response to intervention with anthocyanin-rich foods. These investigations would better inform nutritionists working with IBD patients in real-world diet intervention scenarios to promote gut homeostasis, suppress inflammation and potentially reduce the risk of CAC.

## Data Availability

Supporting sequencing data for this manuscript are available to the public at Utah State University Digital Commons repository, https://doi.org/10.26078/ats5-4m77 (accessed on 8 November 2022). Available files for each experiment include .txt mapping files with sample attribute information,.csv files with 16S rRNA sequence count data with either OTU or ASV identifiers, and .csv files with taxonomy mapped to either OTU or ASV identifiers. All other data are contained within the article and accompanying Supplementary Material.

## Supplementary Materials

The following supporting information can be downloaded at: https://www.mdpi.com/article/10.3390/nu14245270/s1, Figure S1: Diagram depicting design of the pilot study (experiment A) and the black raspberry supplementation with standard and Western basal diets study (experiment B); Figure S2: Food and energy intake, final body weight, final lean and fat mass, and glucose tolerance; Figure S3: Rarefaction curve analysis by experimental group and time point (experiment A); Figure S4: Taxonomic classification of mouse fecal bacteria (experiment A); Figure S5: Relative abundance of fecal microbiome at the family taxonomic level (experiment B); Figure S6: Alpha diversity of mouse fecal microbiomes for each experimental time point (experiment A); Figure S7: Food and energy intake over the study period (experiment A); Figure S8: Relative liver, kidney, spleen and cecum content weight (experiment B); Figure S9: Rarefaction curve analysis by experimental group and time point (experiment B); Figure S10: Relative abundance of selected bacteria families of interest and the ratio of Firmicutes to Bacteroidetes over the study time points (experiment B); Figure S11: Fecal microbiome community structures depicted as heat trees showing the relative abundance ratios for comparisons across study time points within each experimental diet group (experiment B); Figure S12: Beta diversity of mouse fecal microbiomes over each time point within each experimental diet group (experiment B); Figure S13: Metagenome predicted functions classified using KEGG metabolism orthology with tax4fun (experiment B); Figure S14: Longitudinal analysis of fecal microbiome taxonomy and functional capacity (experiment B) for basal diets without BRB supplementation (AIN/CON and TWD/CON) groups. Table S1: Experimental diet formulations; Table S2: Alpha diversity pairwise comparisons for effects of time point only, irrespective of basal diet or BRB supplement (experiment B); Table S3: Alpha diversity pairwise comparisons by experimental group within time points (experiment B); Table S4: Alpha diversity pairwise comparisons by time point within experimental group (experiment B); Table S5: Short-chain fatty acids pairwise comparisons for effects of time point only, irrespective of basal diet or BRB supplement (experiment B); Table S6: Short-chain fatty acids pairwise comparisons by experimental group within time points (experiment B); Table S7: Short-chain fatty acids pairwise comparisons by time point within experimental group (experiment B); File S1: Experiment A (pilot study) microbiome count data.xlsx; File S2: Experiment B (diet comparison) microbiome count data.xlsx; File S3: Experiment A metagenomeSeq statistics.xlsx; File S4: Experiment B metagenomeSeq statistics.xlsx.
