# Supplementary material for: Dietary Supplementation with Black Raspberries Altered the Gut Microbiome Composition in a Mouse Model of Colitis-Associated Colorectal Cancer, although with Differing Effects for a Healthy versus a Western Basal Diet
Source: Nutrients. 2022 Dec 10;14(24):5270. doi: 10.3390/nu14245270 (PMC9786988; doi:10.3390/nu14245270)
Supplement: Supplementary file 1 [file nutrients-14-05270-s001.zip › nutrients-2085245-supplementary.pdf]

nutrients

Supplementary Material

Dietary supplementation with black raspberries altered the gut microbiome composition in a mouse model of colitis-associated colorectal cancer, although with differing effects for a healthy versus a Western basal diet

Daphne M. Rodriguez, Korry J. Hintze, Giovanni Rompato, Arnaud J. Van Wettere, Robert E. Ward, Sumira Phatak, Canyon Neal, Tess Armbrust, Eliza Stewart, Aaron Thomas , and Abby D. Benninghoff *

* Correspondence: abby.benninghoff@usu.edu; Tel.: +01-435-797-8649

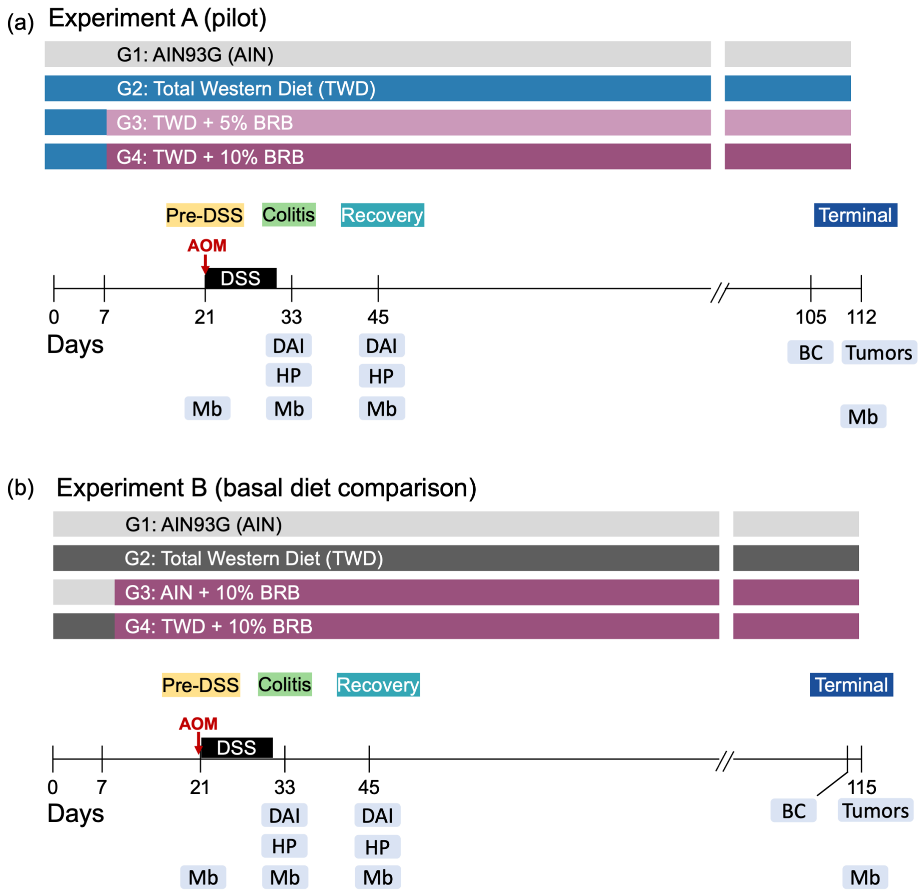

Figure S1. Diagram depicting design of the pilot study (experiment A) and the black raspberry supplementa-tion with standard and Western basal diets study (experiment B). (**a**) For experiment A, basal diets are rep-resented as gray (AIN) or blue (TWD) bars with supplementation of low concentration black raspberry (5% BRB) and high concentration (10% BRB) depicted as light and dark pink, respectively. Experimental time points include pre-DSS (day 21), colitis (day 33), recovery (day 45) and terminal (day 112). Endpoints measured are indicated below the timeline, including the disease activity index (DA), histopathology (HP), fecal microbiome profiling (Mb), body composition (BC) and colon tumors. (**b**) For experiment B, basal diets AIN and TWD are represented as light gray or dark grey bars, respectively, with 10% black raspberry sup-plementation shown as dark pink. Time points are as for experiment A, except for tumors assessed at day 115. Endpoints are indicated below the timeline. Other abbreviations are as follows: azoxymethane, AOM; dextran sodium sulfate, DSS.

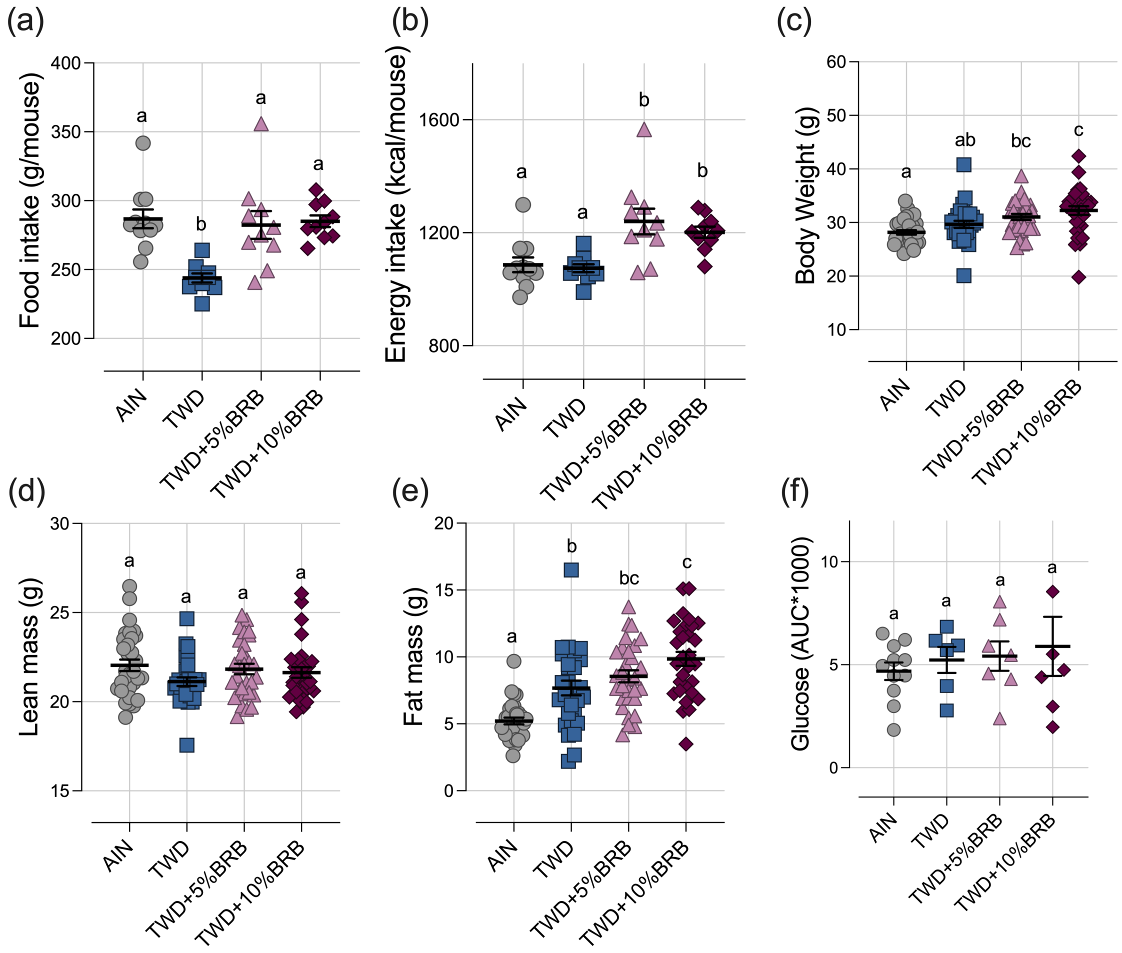

Figure S2. Food and energy intake, final body weight, final lean and fat mass, and glucose tolerance. Food (**a**) and energy (**b**) intakes per cage (*n*=10-11) for the study period are shown for each individual mouse with the mean ± SD. Final body weight (**c**), lean mass (**d**), and fat mass (**e**) values are shown for each individual mouse (*n*=29-32) with the mean ± SE. (**f**) Glucose tolerance expressed as the area under the curve (AUC) determined at the terminal time point shown for individual mice (*n*=6 to 11) with the mean ± SE. Different letters indicate groups are significantly different (p<0.05) as outlined in Materials and Methods.

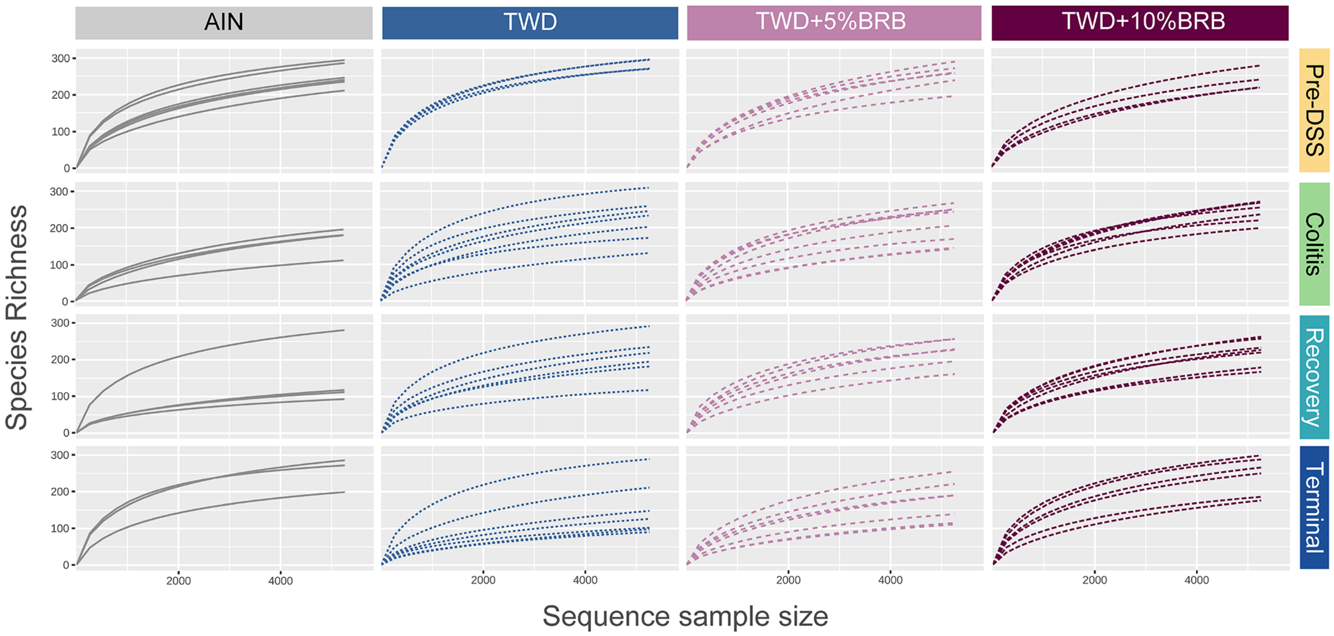

Figure S3. Rarefaction curve analysis by experimental group and time point (experiment A). Curves plot species richness as a function of sequence sample size. For comparisons across experimental groups, data were rarefied to ~5,500 sequences, the lowest total among all the samples.

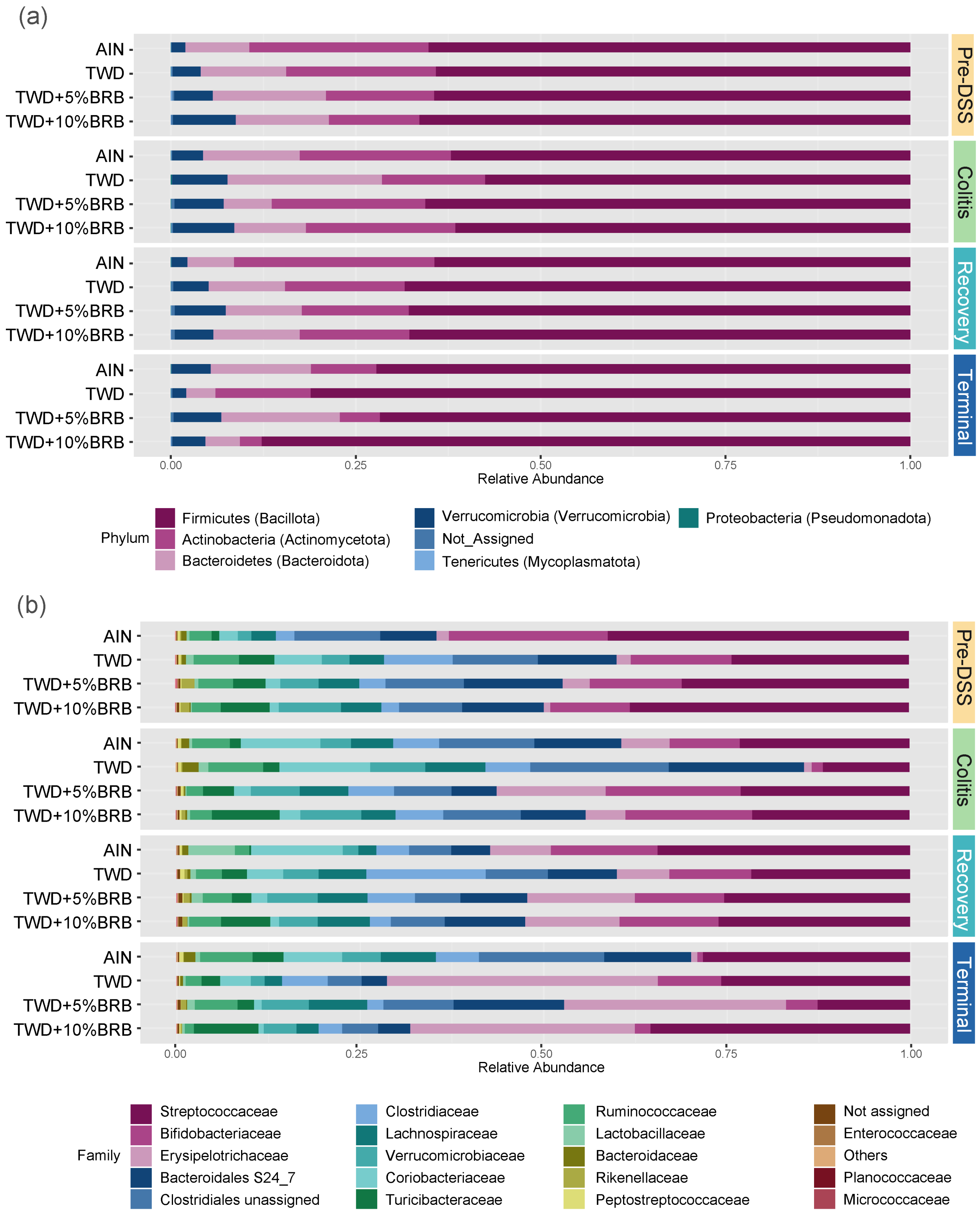

Figure S4. Taxonomic classification of mouse fecal bacteria (experiment A). Data shown are the relative normalized abundance of bacteria annotated to phylum (**a**) or family (**b**) taxonomic levels for the most abundant taxa for each experimental group for each experimental time point. New phylum level taxonomic designations are indicated in parentheses (a).

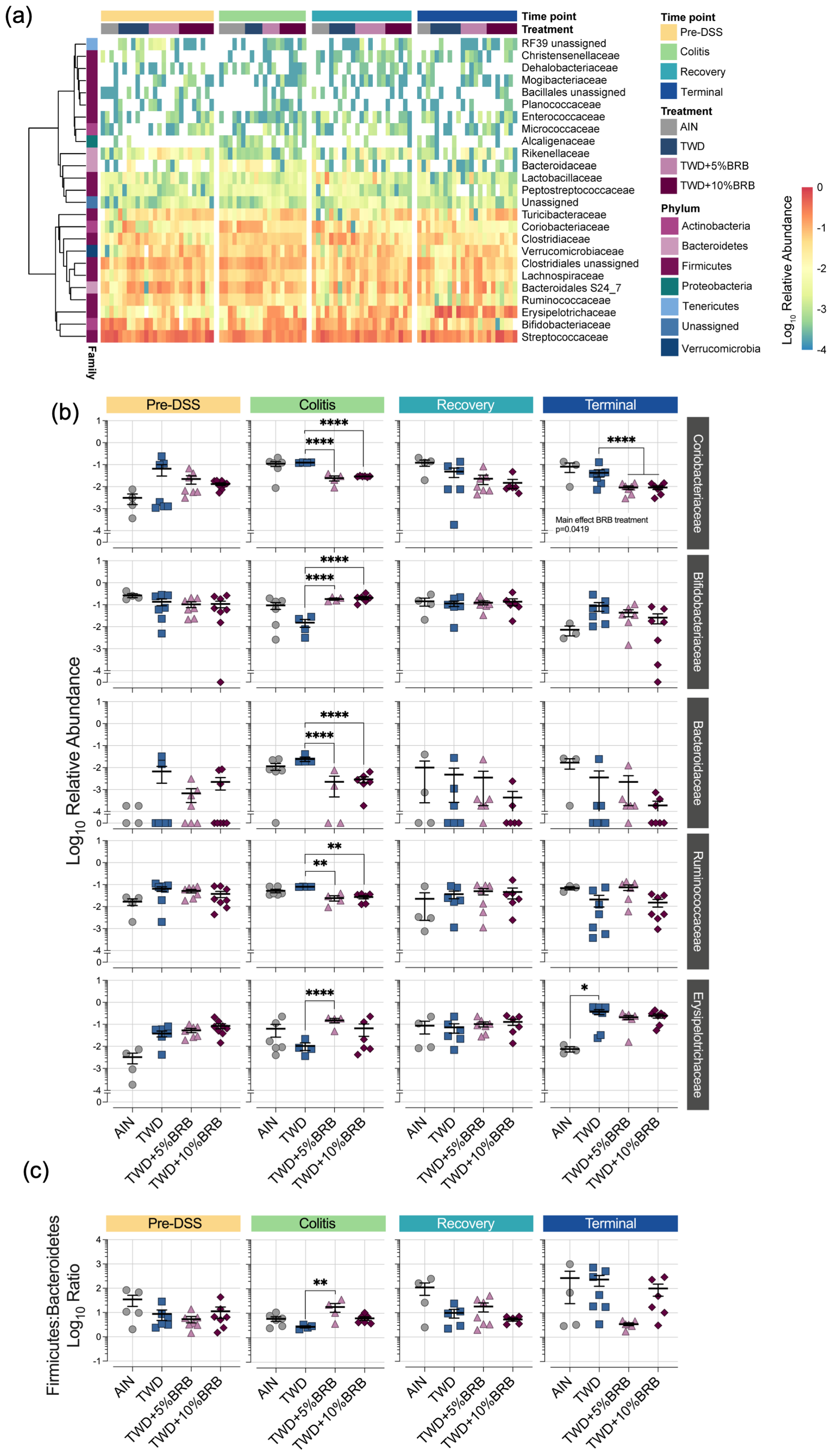
(a)
Time point
Treatment
RF39 unassigned
Christensenellaceae
Dehalobacteriaceae
Mogibacteriaceae
Bacillales unassigned
Planococcaceae
Enterococcaceae
Micrococcaceae
Alcaligenaceae
Rikenellaceae
Bacteroidaceae
Lactobacillaceae
Peptostreptococcaceae
Unassigned
Turicibacteraceae
Coriobacteriaceae
Clostridiaceae
Verrucomicrobiaceae
Clostridiales unassigned
Lachnospiraceae
Bacteroidales S24_7
Ruminococcaceae
Erysipelotrichaceae
Bifidobacteriaceae
Streptococcaceae
Family
Time point
Pre-DSS
Colitis
Recovery
Terminal
Treatment
AIN
TWD
TWD+5%BRB
TWD+10%BRB
Phylum
Actinobacteria
Bacteroidetes
Firmicutes
Proteobacteria
Tenericutes
Unassigned
Verrucomicrobia
Log10 Relative Abundance
(b)
Pre-DSS
Colitis
Recovery
Terminal
Log10 Relative Abundance
Coriobacteriaceae
Bifidobacteriaceae
Bacteroidaceae
Ruminococcaceae
Erysipelotrichaceae
Main effect BRB treatment p=0.0419
AIN
TWD
TWD+5%BRB
TWD+10%BRB
(c)
Pre-DSS
Colitis
Recovery
Terminal
Firmicutes:Bacteroidetes Log10 Ratio
AIN
TWD
TWD+5%BRB
TWD+10%BRB

Figure S5. Relative abundance of fecal microbiome at the family taxonomic level (experiment A). (**a**) Unsupervised hierarchical cluster analysis of the bacteria families compromising a least 1% of fecal microbiome. The heatmap was constructed with clustering by taxa using the Euclidean distance with average clustering, and the color scale represents the log_{10} relative abundance. (**b**) Relative abundance of select bacteria families of interest for each experimental time point, including Coriobacteriaceae, Bifidobacteriaceae, Bacteroidaceae, Ruminococcaceae, and Erysipelotrichaceae. (**c**) Ratio of Firmicutes:Bacteroidetes by experimetnal time point. Ratios were determined using normalized count data for each phylum. For (b) and (c), data are shown as individual values that represent each cage (as the biological unit) with mean ± SE. *, $p<0.05$; **, $p<0.01$; and ****, $p<0.0001$ as outlined in Materials and Methods. Complete results of all metagenomeSeq statistical analyses, including selected pairwise comparisons by experimental group, are provided in File S3.

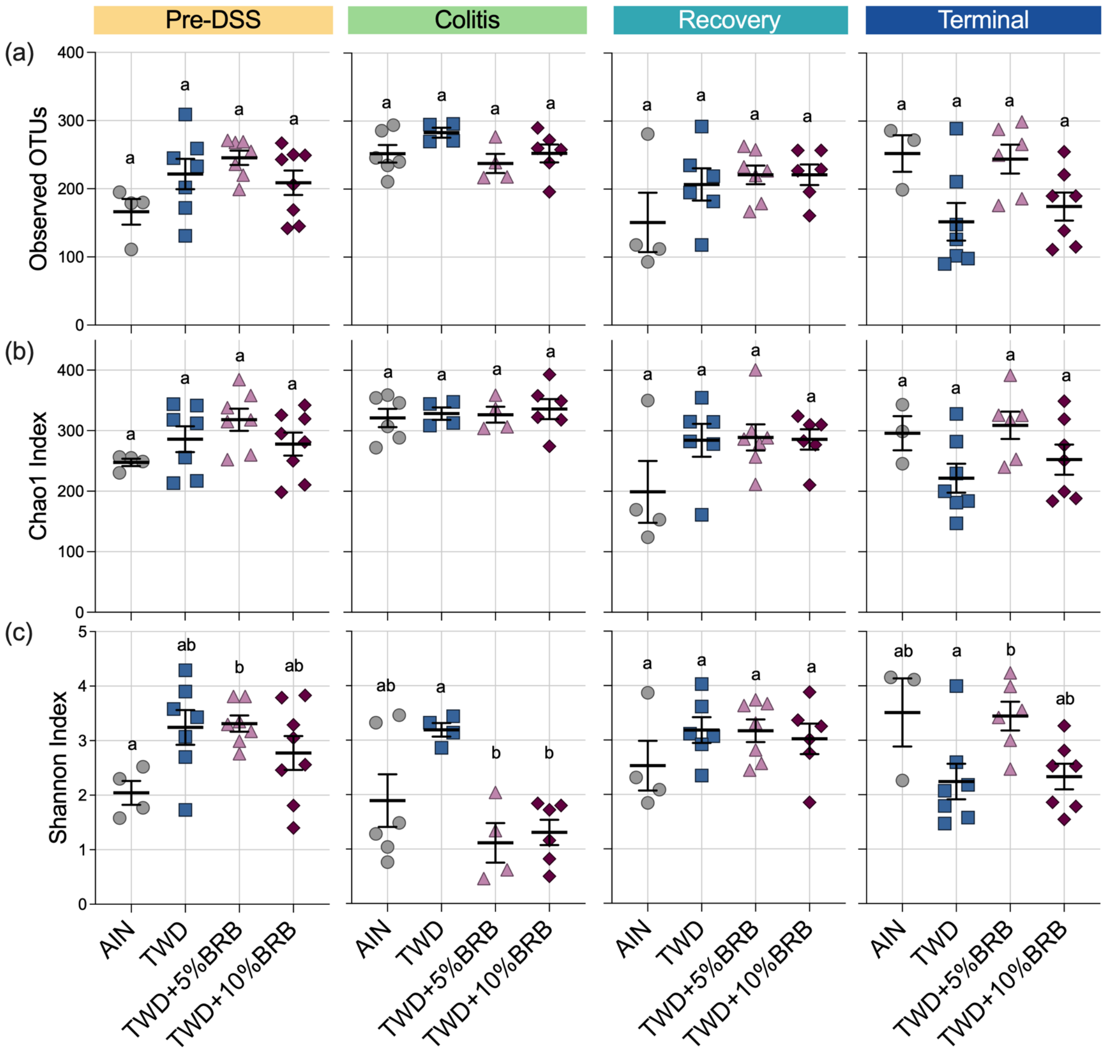

Figure S6. Alpha diversity of mouse fecal microbiomes for each experimental time point (experiment A). Alpha diversity measures include (**a**) observed OTUs, (**b**) the Chao1 index, and (**c**) the Shannon index. Data are shown as individual values representing each cage (as the biological unit) with mean ± SE. Inset tables show the statistical model main effects including all experimental factors for each α-diversity measure. Different letters indicate groups are significantly different (p<0.05) as outlined in Materials and Methods.

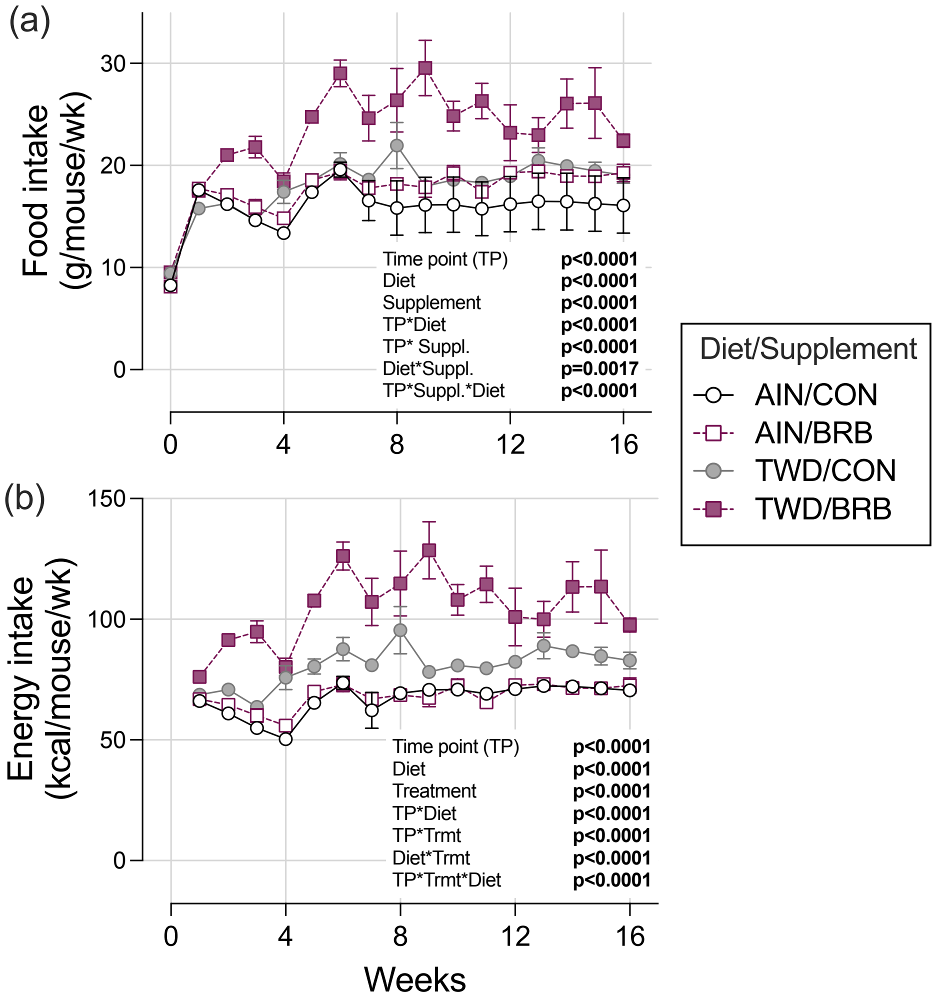

Figure S7. Food and energy intake over the study period (experiment B). Values shown are the average estimated food intake (**a**) and energy intake (**b**) per mouse per week ± SE. Inset tables show the statistical model main effects for time point (TP), basal diet (Diet), and BRB treatment (Trmt) and all possible interactions determined by statistical methods outlined in Materials and Methods.

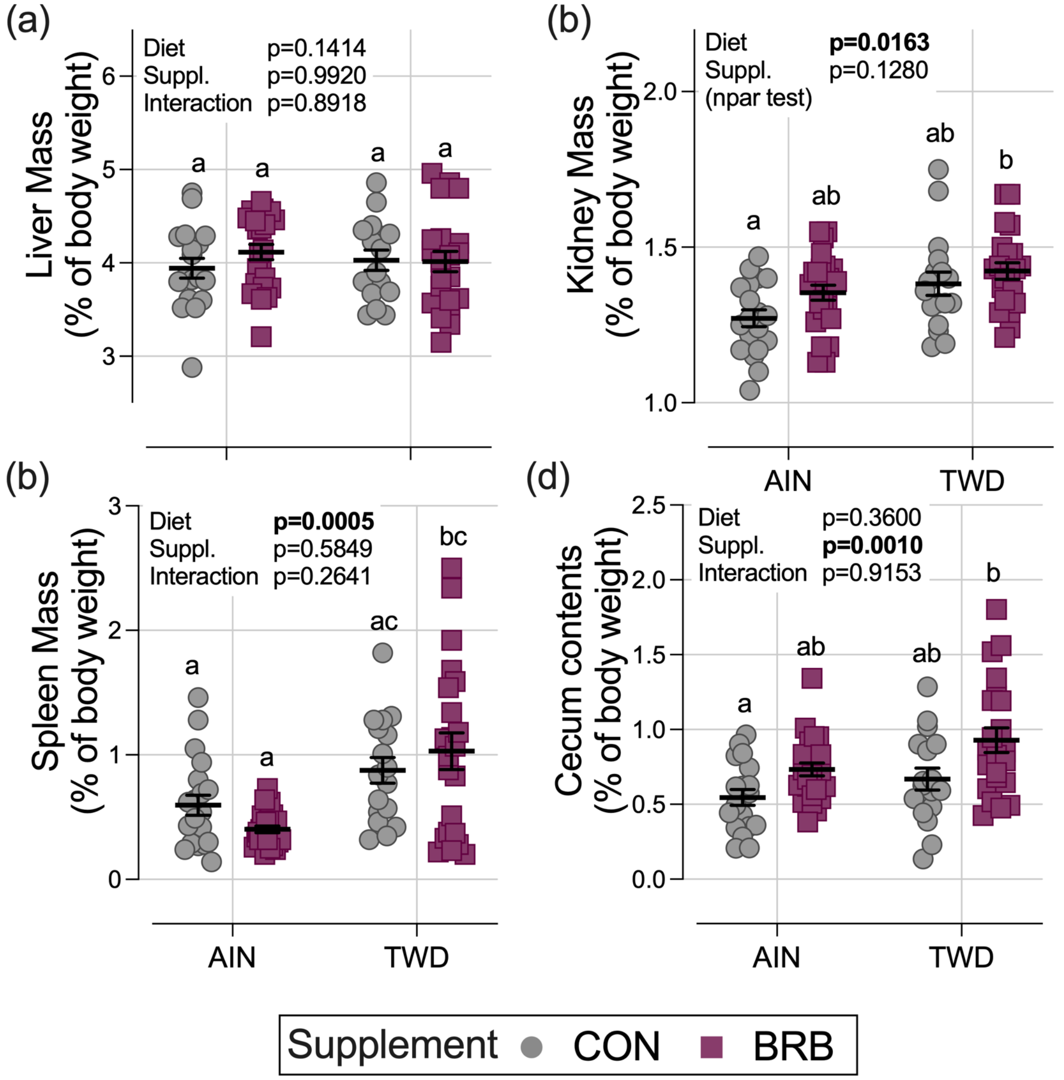

Figure S8. Relative liver, kidney, spleen, and cecum content weights (experiment B). Data for liver (**a**), kidney (**b**), spleen (**c**), and cecum content (**d**) weights are shown as a proportion of the final body weight. Values for individual mice (*n*=17-25) are shown with mean ± SE. Inset tables provide the model main effects for diet, treatment, and their interaction or "npar test" if a nonparametric test was required, and different letters indicate groups are significantly different (p<0.05) as determiend by statistical methods outlined in Materials and Methods.

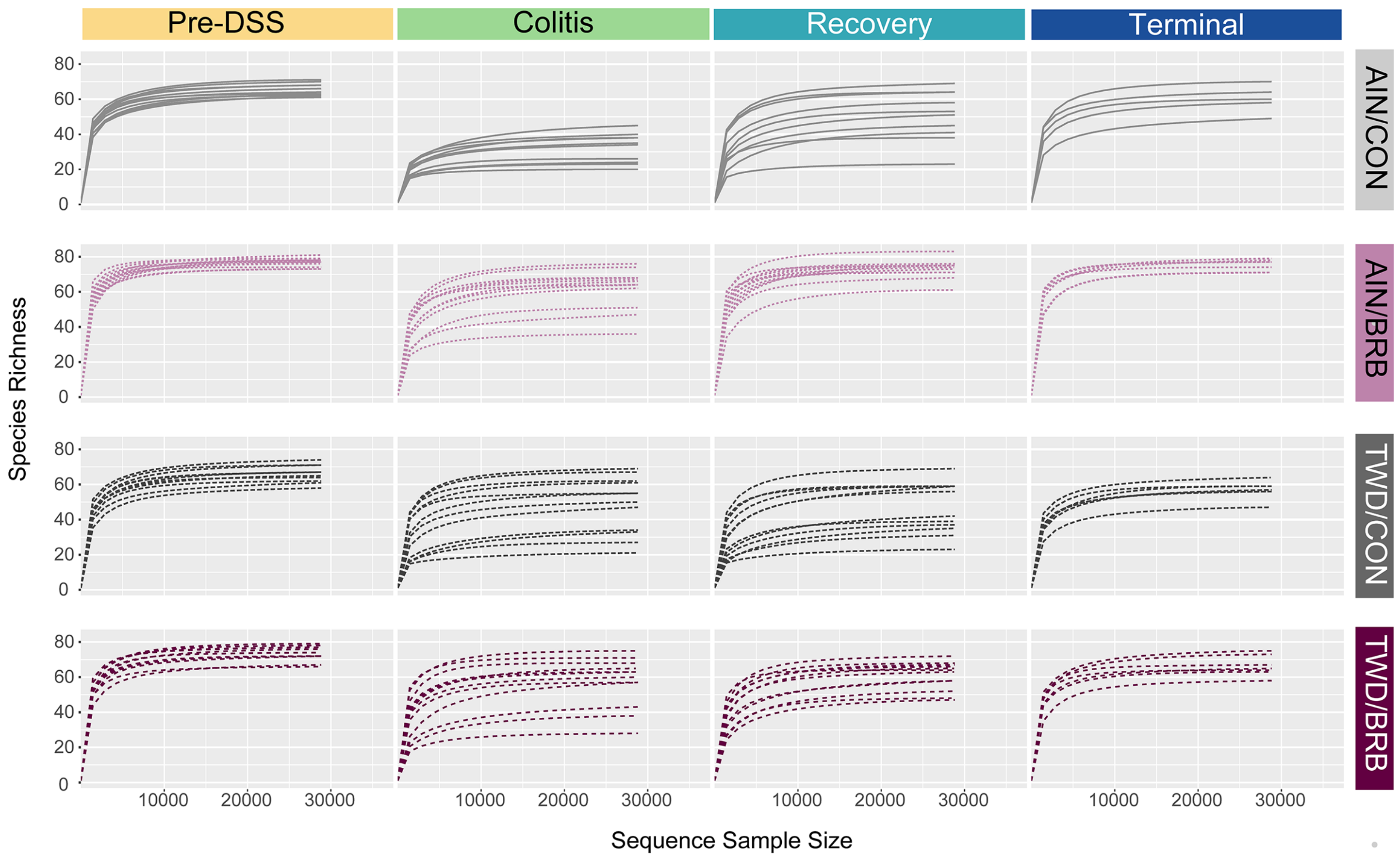

Figure S9. Rarefaction curve analysis by experimental group and time point (experiment B). Curves plot species richness as a function of sequence sample size. For comparisons across experimental groups, data were rarefied to 28,909 sequences, the lowest total among all the samples. These curves indicate that saturation was reached satisfactorily for most samples and that additional sequence reads were unlikely to substantially increase the number of species detected.

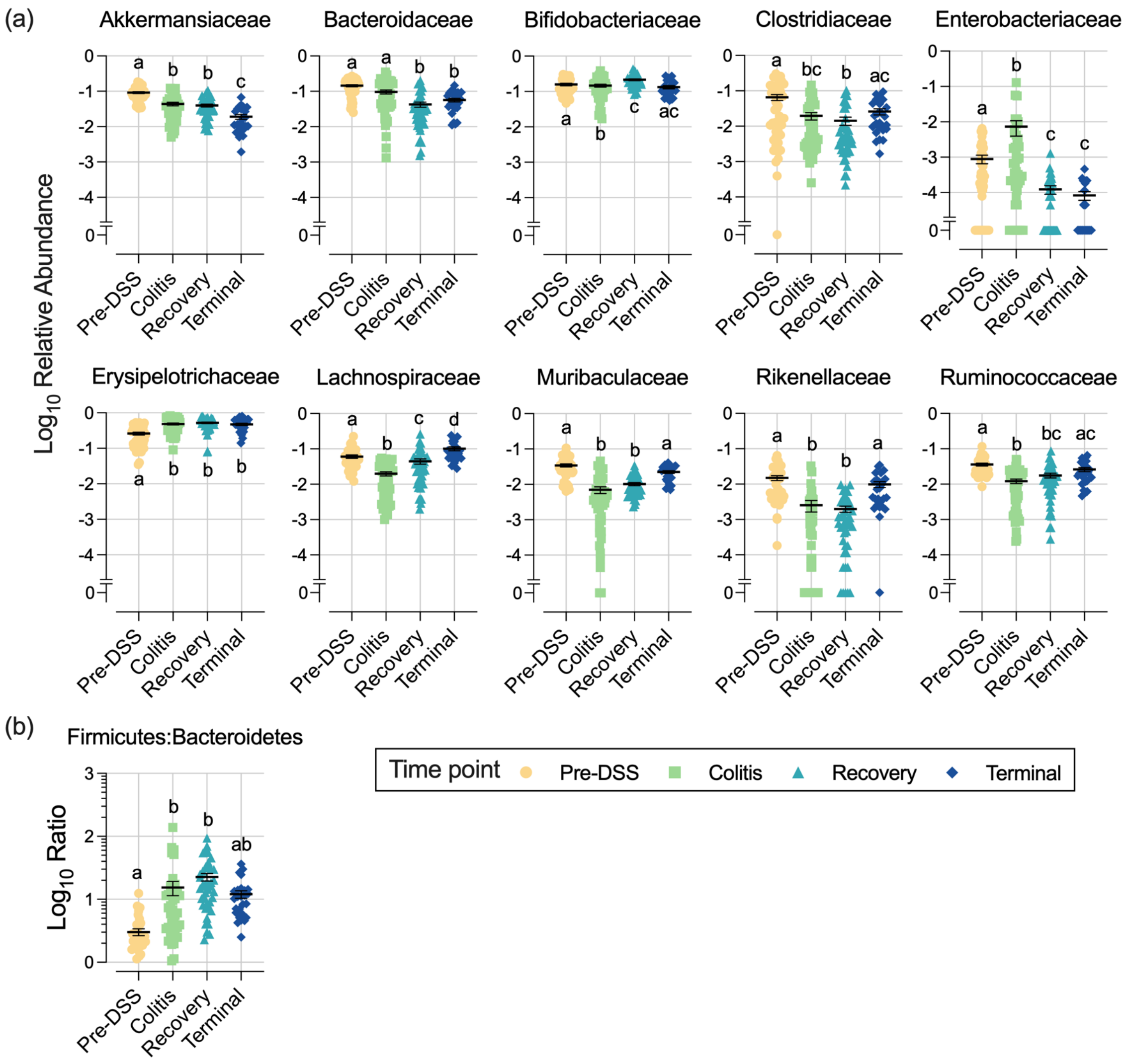

Figure S10. Relative abundance of selected bacteria families of interest and the ratio of Firmicutes-to-Bacteroidetes over the study time points (experiment B). The $\log_{10}$ relative abundance values for selected taxa (**a**) and the $\log_{10}$ ratio values for Firmicutes-to-Bacteroidetes (**b**) are shown, irrespective of basal diet or BRB supplementation, for each experimental time point for individual mice with the mean ± SE. Different letters indicate that relative abundances or ratios for time points are significantly different ($p<0.05$) as determined by statistical methods outlined in Materials and Methods. Complete results of all metagenomeSeq statistical analyses are provided in File S4.

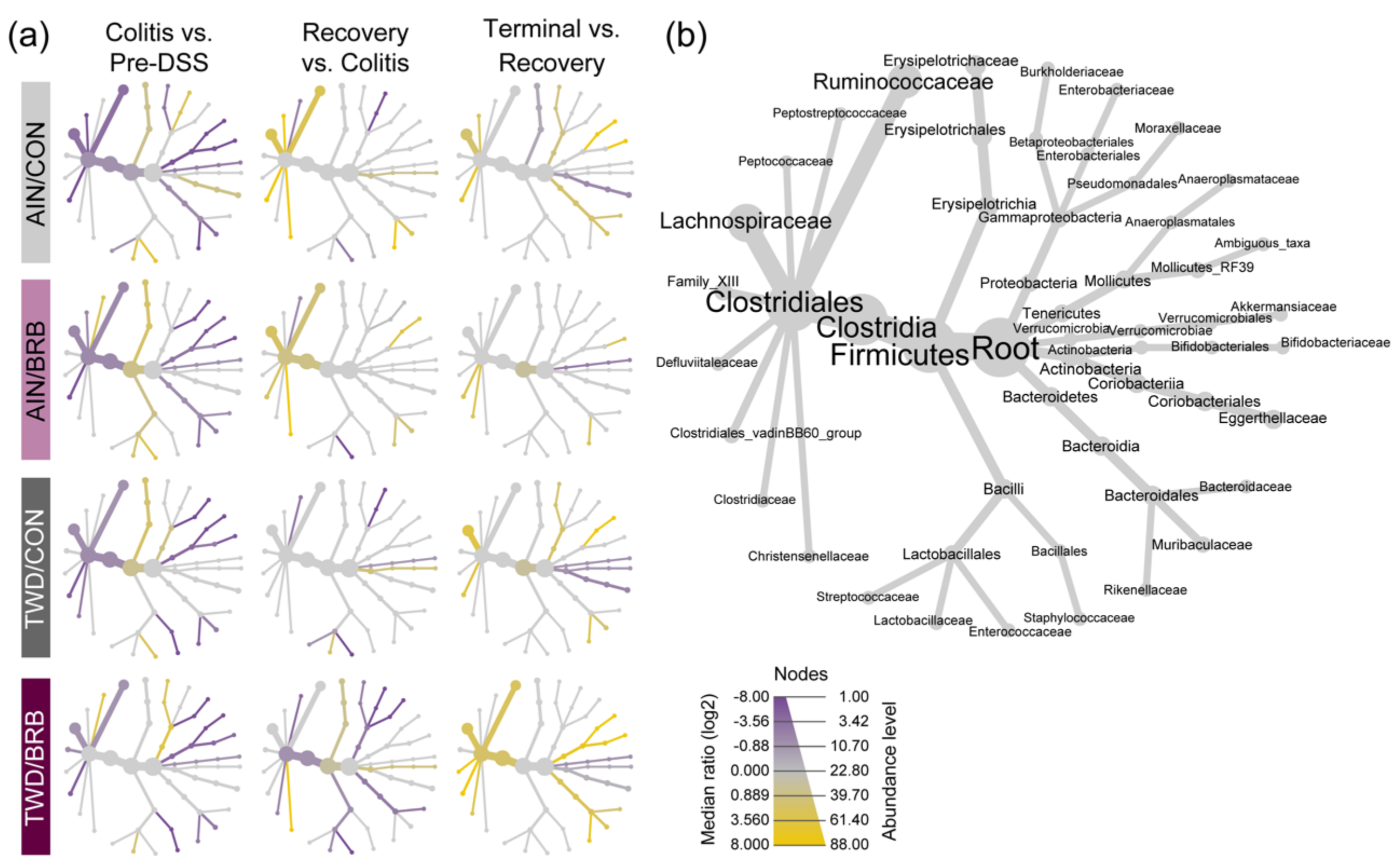

Figure S11. Fecal microbiome community structures depicted as heat trees showing the relative abundance ratios for comparisons across study time points within each experimental diet group (expermient B). The heat tree analysis leverages the hierarchical structure of taxonomic classifications to quantitatively (using the median abundance) and statistically (using the non-parametric Wilcoxon Rank Sum test) depict taxonomic differences between microbial communities. (**a**) Comparisons of colitis vs. pre-DSS, recovery vs. colitis, and terminal vs. recovery time points with yellow indicating increase abundance with disease progression (advancing time point) and purple indicating reduced abundance as indicated by the scale. For example, relative abundance of Ruminococcaceae is increased (yellow) at recovery vs. colitis time point for the AIN/CON experimental diet group, whereas Enterobacteriaceae is less abundant (purple). (**b**) Phylogenetic structure of fecal microbiome bacteria community.

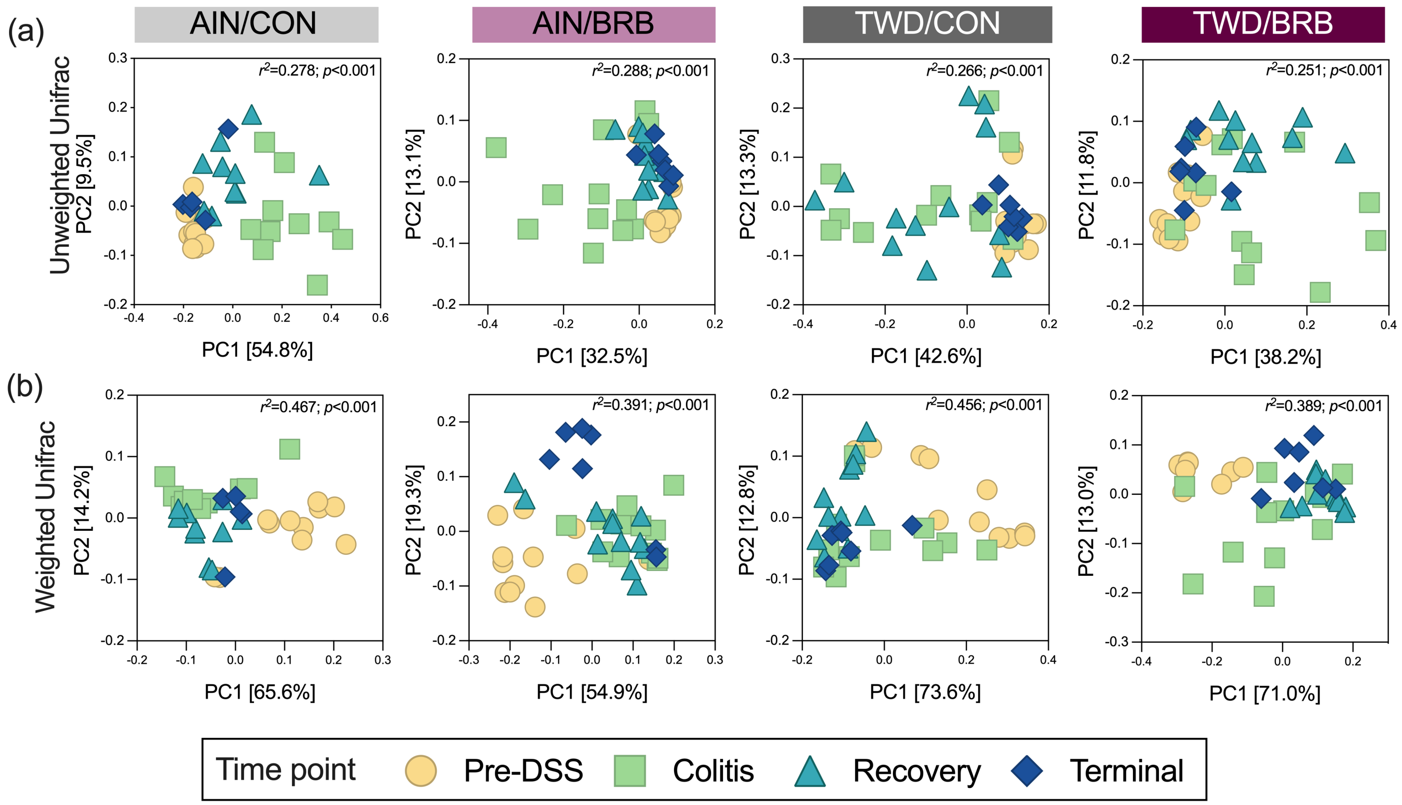

Figure S12. Beta diversity of mouse fecal microbiomes over each time point within each experimental diet group (experiment B). Principal coordinate plots depicting fecal microbiome beta diversity using (a) unweighted or (b) weighted unifrac distances are shown using the first two components. The variation attributed to PC1 and PC2 are shown along with the r2 and permanova p-values for each plot.

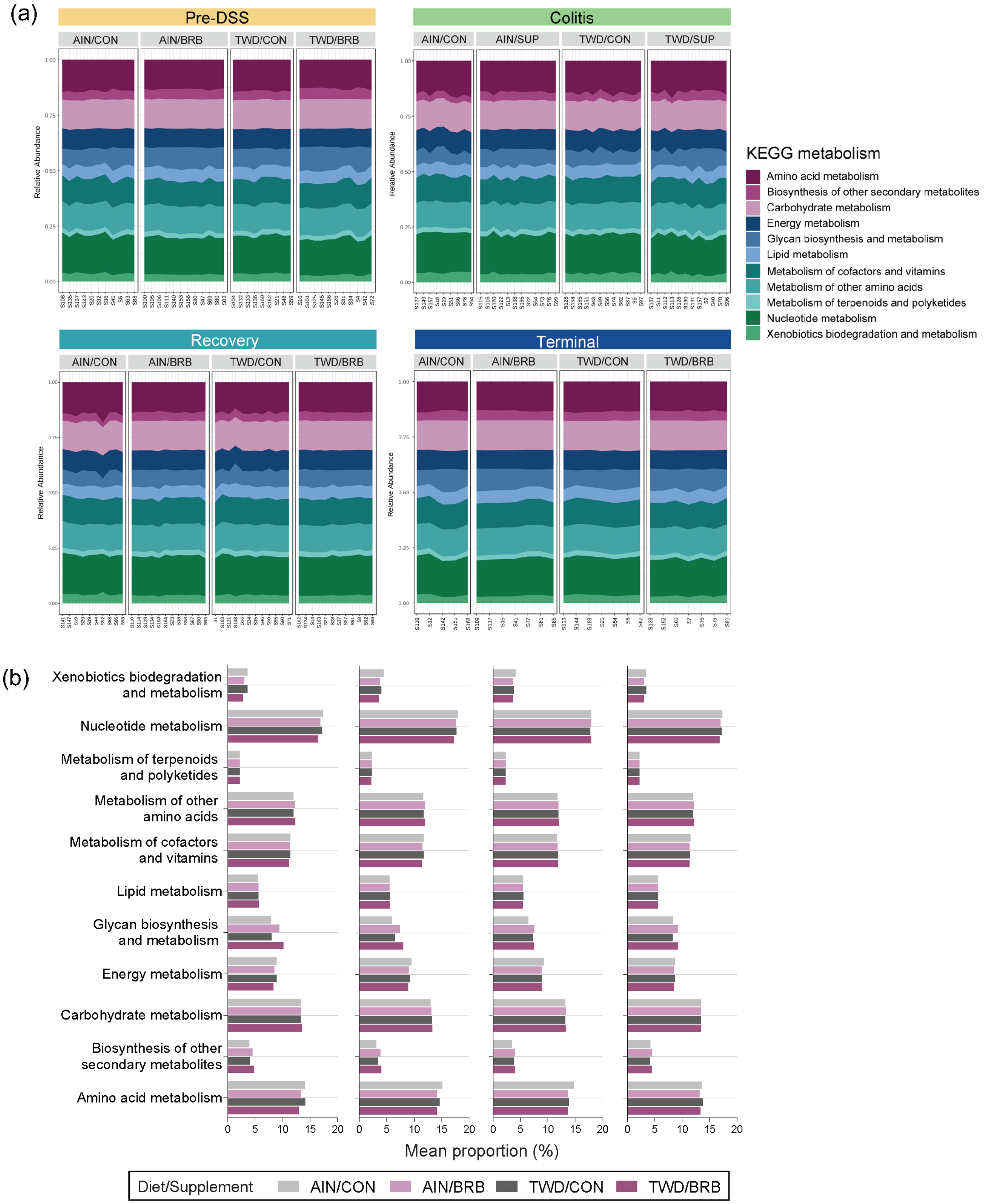

Figure S13. Metagenome predicted functions classified using KEGG metabolism orthology with tax4fun (experiment B). (a) Stacked area plots show the total hits normalized by category size for KEGG level 1 metabolism terms. (b) Mean proportions (%) for KEGG metabolism categories for each experimental diet group.

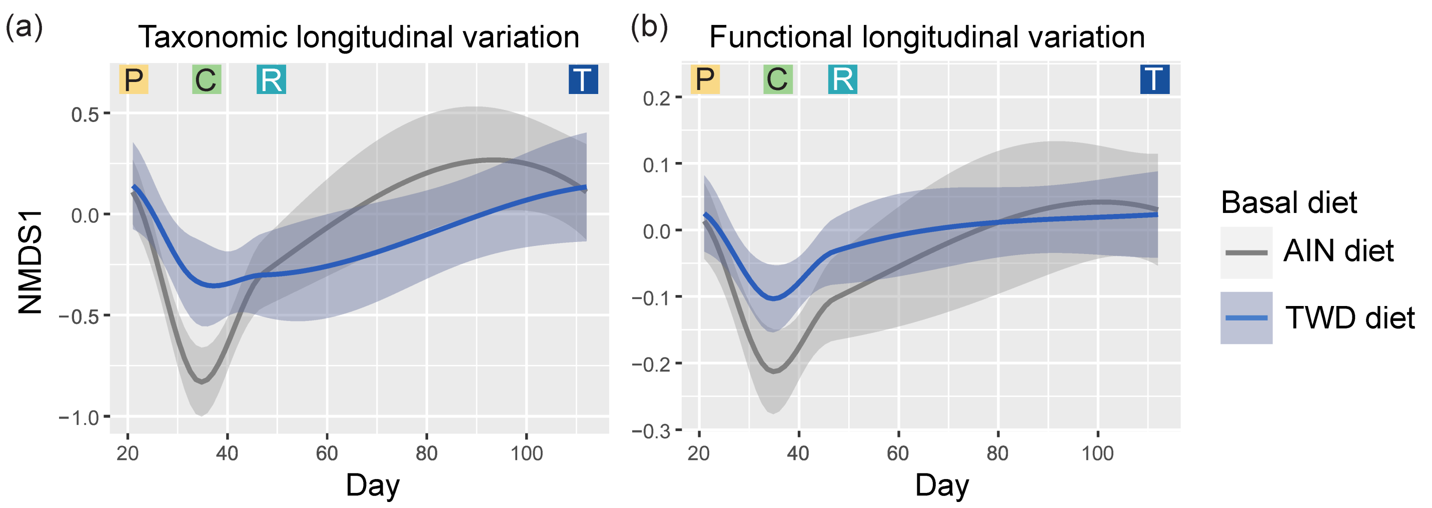

Figure S14. Longitudinal analysis of fecal microbiome taxonomy and functional capacity (experiment B) for AIN and TWD basal diets without BRB supplementation (AIN/CON and TWD/CON groups). Longitudinal variation is shown as the first dimention of Bray-Curtis dissimilarity beta-diversity for taxonomy based on ASV abundances (**a**) or function based on KEGG term abundances (**b**). Loess-smoothed trajectories of sample microbiomes from each experimental group are plotted with gray areas representing the 95% confidence interval. P, pre-DSS; C, colitis; R, recovery; and T, terminal time points.

Table S1. Experimental diet formulations

			AIN93G	AIN93G + 10% BRB	TWD	TWD + 10% BRB
Energy density (kcal/g)			3.76	3.77	4.35	4.34
**Macronutrients**	Carbohydrates (g/kg diet)	Black Raspberry Powder		100		100
		Corn Starch	397.5	374.3	230	201
		Maltodextrin	132	132	70	70
		Sucrose	100	73.2	261.2	232.8
		Cellulose	50	12.5	30	
	Kcal (% of total)		*63.9%*	*63.9%*	*50.0%*	*49.8%*
	Proteins (g/kg)	Casein	200	187.5	190	177.5
		L-cystine	3	3	2.9	2.9
	Kcal (% of total)		*18.8%*	*18.9%*	*15.5%*	*15.6%*
	Fats (g/kg)	Soybean oil	70	70	190	177.5
		Anhydrous milk fat			36.3	36.3
		Olive Oil			28	28
		Lard			28	28
		Beef tallow			24.8	24.8
		Corn oil			16.5	16.5
		Cholestrol			0.4	0.4
	Kcal (% of total)		*17.2%*	*17.1%*	*34.5%*	*34.6%*
**Micronutrients**	Minerals (mg/kg)	Calcium	5000	5000	2011	2011
		Phosphorus	3000	3000	2757	2757
		Sodium	1019	1019	7078	7078
		Potassium	3600	3600	5333	5333
		Magnesium	507	507	589	589
		Iron	35	35	31	31
		Zinc	30	30	25	25
		Copper	6	6	2.6	2.6
		Selenium	0.15	0.15	0.2	0.2
	Vitamins (unit/kg)	Thiamin (mg)	5	5	3.5	3.5
		Riboflavin (mg)	6	6	4.4	4.4
		Niacin (mg)	30	30	50.6	50.6
		Pyridoxine (mg)	6	6	3.9	3.9
		Folate (mg)	2	2	1.3	1.3
		Vitamin B12 (μg)	25	25	11	11
		Vitamin A (IU)	4000	4000	4300	4300
		Vitamin D (IU)	1000	1000	391	391
		Vitamin E (IU)	75	75	24.6	24.6
		Vitamin K (μg)	750	750	189	189
		Choline (mg)	1027	1027	648	648

Note: Abbreviations for diets are the following: total Western diet, TWD. Composition of the TWD was published previously.[1] No data are available in NHANES for chloride, manganese, iodine, pantothenic acid, biotin, or ultra-trace minerals. Levels of these components mimics that of the baasal diet to formulate BRB supplemented diets.

[1] Hintze, K.J.; Benninghoff, A.D.; Ward, R.E. Formulation of the total western diet (TWD) as a basal diet for rodent cancer studies. *J. Agric. Food Chem.* **2012**, *60*, 6736-6742, doi:10.1021/jf204509a.

Table S2. Alpha diversity pairwise comparisons for effects of time point only, irrespective of basal diet or BRB supplement (experiment B)

	Alpha Diversity Measure		
**Time point comparison**	**Observed ASVs**	**Chao1 index**	**Shannon Index**
Pre-DSS vs. Colitis	**0.0001**	**0.0001**	**0.0001**
Pre-DSS vs. Recovery	**0.0001**	**0.0001**	**0.0001**
Pre-DSS vs. Terminal	0.2246	0.2742	**0.0008**
Colitis vs. Recovery	**0.0012**	**0.0007**	0.9090
Colitis vs. Terminal	**0.0001**	**0.0001**	**0.0163**
Recovery vs. Terminal	**0.0006**	**0.0007**	**0.0027**

Values shown are the Tukey HSD post-hoc comparison *p*-values for each pairwise comparison between experimental diet groups within each time point following a generalized linear model analysis. Main effects of all experimental factors are presented in Figure 11.

Table S3. Alpha diversity pairwise comparisons by experimental group within time points (experiment B)

		Alpha Diversity Measure		
**Time point**	**Comparison**	**Observed ASVs**	**Chao1 index**	**Shannon Index**
Pre-DSS	AIN/CON vs. AIN/BRB	**0.0001**	**0.0001**	**0.0020**
Pre-DSS	AIN/CON vs. TWD/CON	0.9588	0.9448	0.4599
Pre-DSS	AIN/CON vs. TWD/BRB	**0.0001**	**0.0001**	0.0741
Pre-DSS	AIN/BRB vs. TWD/CON	**0.0001**	**0.0001**	0.0988
Pre-DSS	AIN/BRB vs. TWD/BRB	0.5004	0.5545	0.5540
Pre-DSS	TWD/CON vs. TWD/BRB	**0.0001**	**0.0001**	0.7349
Colitis	AIN/CON vs. AIN/BRB	**0.0001**	**0.0001**	**0.0373**
Colitis	AIN/CON vs. TWD/CON	**0.0386**	**0.0299**	0.5323
Colitis	AIN/CON vs. TWD/BRB	**0.0004**	**0.0003**	**0.0048**
Colitis	AIN/BRB vs. TWD/CON	0.1733	0.1204	0.4408
Colitis	AIN/BRB vs. TWD/BRB	0.9740	0.9295	0.8456
Colitis	TWD/CON vs. TWD/BRB	0.3547	0.3529	0.1079
Recovery	AIN/CON vs. AIN/BRB	**0.0001**	**0.0002**	**0.0001**
Recovery	AIN/CON vs. TWD/CON	0.9294	0.8141	0.4909
Recovery	AIN/CON vs. TWD/BRB	0.1359	0.2147	0.9996
Recovery	AIN/BRB vs. TWD/CON	**0.0001**	**0.0001**	**0.0001**
Recovery	AIN/BRB vs. TWD/BRB	**0.0424**	**0.0444**	**0.0001**
Recovery	TWD/CON vs. TWD/BRB	**0.0251**	**0.0229**	0.3867
Terminal	AIN/CON vs. AIN/BRB	**0.0005**	**0.0010**	0.0915
Terminal	AIN/CON vs. TWD/CON	0.6076	0.5214	0.4618
Terminal	AIN/CON vs. TWD/BRB	0.0811	0.1548	0.9460
Terminal	AIN/BRB vs. TWD/CON	**0.0001**	0.0871	**0.0014**
Terminal	AIN/BRB vs. TWD/BRB	0.0916	**0.0001**	0.1797
Terminal	TWD/CON vs. TWD/BRB	**0.0022**	**0.0037**	0.1464

Values shown are the Tukey HSD post-hoc comparison *p*-values for each pairwise comparison between experimental diet groups within each time point following a generalized linear model analysis.

Table S4. Alpha diversity pairwise comparisons by time point within experimental group (experiment B)

		Alpha Diversity Measure		
**Diet/Treatment**	**Comparison**	**Observed ASVs**	**Chao1 index**	**Shannon index**
AIN/BRB	Pre-DSS vs. Colitis	**0.0001**	**0.0001**	**0.0001**
AIN/BRB	Pre-DSS vs. Recovery	**0.0008**	0.1908	0.0831
AIN/BRB	Pre-DSS vs. Terminal	0.7791	1.0000	0.9157
AIN/BRB	Colitis vs. Recovery	**0.0011**	**0.0005**	0.0871
AIN/BRB	Colitis vs. Terminal	**0.0001**	**0.0001**	**0.0054**
AIN/BRB	Recovery vs. Terminal	0.0758	0.3309	0.4702
AIN/CON	Pre-DSS vs. Colitis	**0.0001**	**0.0001**	**0.0001**
AIN/CON	Pre-DSS vs. Recovery	0.1916	**0.0017**	**0.0007**
AIN/CON	Pre-DSS vs. Terminal	1.0000	0.8782	0.7015
AIN/CON	Colitis vs. Recovery	**0.0006**	**0.0003**	0.6517
AIN/CON	Colitis vs. Terminal	**0.0001**	**0.0001**	**0.0106**
AIN/CON	Recovery vs. Terminal	0.3235	0.0799	0.0978
TWD/BRB	Pre-DSS vs. Colitis	**0.0098**	**0.0005**	0.0549
TWD/BRB	Pre-DSS vs. Recovery	**0.0029**	**0.0046**	**0.0001**
TWD/BRB	Pre-DSS vs. Terminal	0.4629	0.6254	0.1714
TWD/BRB	Colitis vs. Recovery	0.9695	0.8556	**0.0404**
TWD/BRB	Colitis vs. Terminal	0.4866	0.0569	0.9976
TWD/BRB	Recovery vs. Terminal	0.2803	0.2263	0.0650
TWD/CON	Pre-DSS vs. Colitis	**0.0004**	**0.0110**	**0.0014**
TWD/CON	Pre-DSS vs. Recovery	**0.0034**	**0.0031**	**0.0001**
TWD/CON	Pre-DSS vs. Terminal	0.6498	0.5255	**0.0017**
TWD/CON	Colitis vs. Recovery	0.8779	0.9658	0.3080
TWD/CON	Colitis vs. Terminal	**0.0458**	0.4471	0.9588
TWD/CON	Recovery vs. Terminal	0.1768	0.2445	0.7434

Values shown are the Tukey HSD post-hoc comparison *p*-values for each pairwise comparison across time points within each experimental diet group following a generalized linear model analysis.

Table S5. Short-chain fatty acids pairwise comparisons for effects of time point only, irrespective of basal diet or BRB supplement (experiment B)

	Alpha Diversity Measure						
**Time point comparison**	**Acetic**	**Butyric**	**Capric**	**Isobutyric**	**Isovaleric**	**Propionic**	**Valeric**
Pre-DSS vs. Colitis	**0.0001**	**0.0001**	0.2944	0.9368	0.6528	**0.0001**	**0.0472**
Pre-DSS vs. Recovery	0.4378	0.8214	0.9956	0.3249	**0.0422**	0.4953	0.6444
Pre-DSS vs. Terminal	0.1946	0.9286	0.9993	0.9417	0.9671	0.6054	0.9998
Colitis vs. Recovery	**0.0001**	**0.0001**	0.1861	0.6898	0.4568	**0.0001**	**0.0012**
Colitis vs. Terminal	**0.0001**	**0.0001**	0.4586	0.7046	0.4512	**0.0001**	0.0816
Recovery vs. Terminal	0.9144	0.5192	0.9869	0.1689	**0.0285**	1.0000	0.7716

Values shown are the Tukey HSD post-hoc comparison *p*-values for each pairwise comparison between experimental diet groups within each time point following a generalized linear model analysis. Main effects of all experimental factors are presented in Figure 15.

Table S6. Short chain fatty acids pairwise comparisons by experimental group within time points (experiment B)

		Short Chain Fatty Acid						
**Time point**	**Comparison**	**Acetic**	**Butyric**	**Capric**	**Isobutyric**	**Isovaleric**	**Propionic**	**Valeric**
Pre-DSS	AIN/BRB vs. AIN/CON	1.0000	0.1788	0.7720	0.9964	0.9987	0.9230	0.2039
Pre-DSS	AIN/CON vs. TWD/CON	0.9943	0.0768	**0.0006**	0.7623	0.3289	0.9928	0.7336
Pre-DSS	AIN/CON vs. TWD/BRB	**0.0001**	**0.0001**	**0.0003**	**0.0023**	**0.0003**	**0.0001**	**0.0015**
Pre-DSS	AIN/BRB vs. TWD/CON	0.9931	0.9779	**0.0051**	0.8768	0.4285	0.8190	0.8000
Pre-DSS	AIN/BRB vs. TWD/BRB	**0.0001**	**0.0001**	**0.0024**	**0.0052**	**0.0007**	**0.0001**	0.2409
Pre-DSS	TWD/BRB vs. TWD/CON	**0.0001**	**0.0001**	0.9977	**0.0389**	0.0524	**0.0001**	**0.0382**
Colitis	AIN/BRB vs. AIN/CON	0.9185	0.9988	0.9614	0.1625	**0.0457**	0.6694	0.0579
Colitis	AIN/CON vs. TWD/CON	0.5982	0.4734	0.9796	**0.0024**	**0.0158**	0.2397	0.0983
Colitis	AIN/CON vs. TWD/BRB	0.4656	0.9061	0.1242	0.8806	0.9439	0.8243	0.3198
Colitis	AIN/BRB vs. TWD/CON	0.2340	0.5319	0.9998	0.2901	0.9519	0.8376	0.9987
Colitis	AIN/BRB vs. TWD/BRB	0.1604	0.8391	0.2637	**0.0429**	**0.0174**	0.9962	0.8983
Colitis	TWD/BRB vs. TWD/CON	0.9951	0.1917	0.2551	**0.0005**	**0.0060**	0.7480	0.9508
Recovery	AIN/BRB vs. AIN/CON	**0.0047**	0.9157	0.0742	**0.0032**	**0.0205**	0.2150	0.9992
Recovery	AIN/CON vs. TWD/CON	0.3944	0.9963	0.0821	**0.0034**	**0.0126**	0.9597	0.9030
Recovery	AIN/CON vs. TWD/BRB	0.9282	0.6859	0.0768	0.9987	0.9868	**0.0152**	0.3071
Recovery	AIN/BRB vs. TWD/CON	0.1997	0.9715	0.9997	1.0000	0.9977	0.0708	0.8408
Recovery	AIN/BRB vs. TWD/BRB	**0.0006**	0.2893	1.0000	**0.0040**	**0.0068**	**0.0001**	0.3552
Recovery	TWD/BRB vs. TWD/CON	0.1261	0.5335	0.9998	**0.0043**	**0.0040**	**0.0467**	0.0747
Terminal	AIN/BRB vs. AIN/CON	0.9999	0.8229	0.4981	0.8417	0.7079	0.9962	0.8788
Terminal	AIN/CON vs. TWD/CON	**0.0204**	0.3536	0.5689	0.1634	0.0520	**0.0019**	0.1227
Terminal	AIN/CON vs. TWD/BRB	0.0951	**0.0001**	0.2772	0.5177	0.7375	**0.0391**	**0.0001**
Terminal	AIN/BRB vs. TWD/CON	**0.0191**	0.8465	**0.0377**	**0.0215**	**0.0025**	**0.0026**	0.4049
Terminal	AIN/BRB vs. TWD/BRB	0.0956	**0.0004**	**0.0142**	0.1410	0.1808	0.0531	**0.0006**
Terminal	TWD/BRB vs. TWD/CON	0.9694	**0.0021**	0.9060	0.9296	0.4350	0.8049	**0.0209**

Values shown are the Tukey HSD post-hoc comparison *p*-values for each pairwise comparison between experimental diet groups within each time point following a generalized linear model analysis.

Table S7. Short-chain fatty acids pairwise comparisons by time point within experimental group (experiment B)

		**Short Chain Fatty Acid**						
**Time point**	**Comparison**	**Acetic**	**Butyric**	**Capric**	**Isobutyric**	**Isovaleric**	**Propionic**	**Valeric**
AIN/CON	Colitis vs. Pre-DSS	**0.0001**	**0.0001**	0.8603	0.9785	1.0000	**0.0004**	0.9763
AIN/CON	Pre-DSS vs. Recovery	0.0859	0.9540	**0.0177**	**0.0035**	**0.0010**	0.7319	0.9799
AIN/CON	Pre-DSS vs. Terminal	0.9858	0.9966	0.6435	1.0000	0.9974	0.9982	0.9447
AIN/CON	Colitis vs. Recovery	**0.0004**	**0.0005**	0.0759	**0.0014**	**0.0013**	**0.0115**	0.8647
AIN/CON	Colitis vs. Terminal	**0.0001**	**0.0010**	0.9565	0.9913	0.9972	**0.0015**	0.9973
AIN/CON	Recovery vs. Terminal	0.3048	0.9949	0.3424	**0.0126**	**0.0092**	0.7187	0.8187
AIN/BRB	Colitis vs. Pre-DSS	**0.0001**	**0.0001**	0.5511	0.1450	**0.0017**	**0.0001**	0.1855
AIN/BRB	Pre-DSS vs. Recovery	0.9153	0.9965	0.9993	0.9963	0.9023	1.0000	0.8997
AIN/BRB	Pre-DSS vs. Terminal	0.9359	0.9996	0.9580	0.7819	0.8089	0.9945	0.8508
AIN/BRB	Colitis vs. Recovery	**0.0001**	**0.0001**	0.6479	0.0910	**0.0130**	**0.0001**	**0.0394**
AIN/BRB	Colitis vs. Terminal	**0.0001**	**0.0001**	0.3309	**0.0264**	**0.0003**	**0.0001**	**0.0493**
AIN/BRB	Recovery vs. Terminal	0.6468	0.9911	0.9297	0.8775	0.4388	0.9941	0.9979
TWD/CON	Colitis vs. Pre-DSS	**0.0001**	**0.0001**	0.6908	**0.0050**	0.0501	**0.0001**	**0.0024**
TWD/CON	Pre-DSS vs. Recovery	0.7001	0.9970	0.0690	0.8876	0.9232	0.7206	0.6337
TWD/CON	Pre-DSS vs. Terminal	**0.0077**	0.9857	0.9813	0.6796	0.6126	0.1069	0.9305
TWD/CON	Colitis vs. Recovery	**0.0001**	**0.0001**	0.4705	**0.0006**	**0.0101**	**0.0001**	**0.0001**
TWD/CON	Colitis vs. Terminal	**0.0001**	**0.0001**	0.9104	0.1426	0.5935	**0.0026**	**0.0222**
TWD/CON	Recovery vs. Terminal	0.0957	0.9504	0.1951	0.2899	0.2814	0.5382	0.3105
TWD/BRB	Colitis vs. Pre-DSS	**0.0001**	**0.0050**	0.4426	**0.0007**	**0.0111**	**0.0270**	0.9987
TWD/BRB	Pre-DSS vs. Recovery	0.9967	**0.0021**	0.9136	0.8813	0.5796	0.8995	0.8928
TWD/BRB	Pre-DSS vs. Terminal	0.9997	0.7847	0.9999	0.5666	0.4994	1.0000	0.9289
TWD/BRB	Colitis vs. Recovery	**0.0001**	**0.0001**	0.1644	**0.0001**	**0.0004**	0.1258	0.8395
TWD/BRB	Colitis vs. Terminal	**0.0001**	0.1524	0.6239	0.0942	0.4701	0.0860	0.9670
TWD/BRB	Recovery vs. Terminal	0.9998	**0.0009**	0.9287	0.2365	0.0781	0.9470	0.6293

Values shown are the Tukey HSD post-hoc comparison p-values for each pairwise comparison between experimental diet groups within each time point following a generalized linear model analysis.
